# Differential involvement of feedback and feedforward control networks across disfluency types in adults who stutter: Evidence from resting state functional connectivity

**DOI:** 10.1371/journal.pone.0333205

**Published:** 2025-09-26

**Authors:** Hannah P. Rowe, Saul A. Frankford, Jackie S. Kim, Jason A. Tourville, Alfonso Nieto-Castanon, Frank H. Guenther

**Affiliations:** 1 Department of Communication Sciences and Disorders, Northeastern University, Boston, Massachusetts, United States of America; 2 Department of Speech, Language and Hearing Sciences, Boston University, Boston, Massachusetts, United States of America; 3 Department of Speech, Language, and Hearing, University of Texas Dallas, Dallas, Texas, United States of America; 4 Department of Biomedical Engineering, Boston University, Boston, Massachusetts, United States of America; 5 MIT, Picower Institute for Learning and Memory, Cambridge, Massachusetts, United States of America; 6 Harvard/MIT, Speech and Hearing Bioscience and Technology Program, Cambridge, Massachusetts, United States of America; National Taiwan Normal University, TAIWAN

## Abstract

**Purpose:**

This study investigated the relationship between different disfluency types (i.e., repetitions, prolongations, and blocks) and resting state functional connectivity in the feedback (FB) and feedforward (FF) control networks in 20 adults who stutter.

**Methods:**

Frequency of each disfluency type was coded in speech samples derived from the Stuttering Severity Instrument, and functional connectivity between brain regions of interest was derived from resting state functional magnetic resonance imaging scans. We used LASSO regressions to identify the connections that most strongly predicted each disfluency type.

**Results:**

Both repetitions and prolongations were significantly associated with increased connectivity in *left ventral motor cortex – right ventral premotor cortex*, which is hypothesized to be involved in FB control of speech. In contrast, blocks were significantly associated with reduced connectivity in *right anterior cerebellum – left ventral lateral thalamic nucleus* and increased connectivity in *left presupplementary motor area – left posterior inferior frontal sulcus*, both of which are hypothesized to be involved in FF control of speech.

**Conclusion:**

Our findings suggest that repetitions and prolongations may be associated with increased reliance on FB-based corrective mechanisms, whereas blocks may be associated with disrupted FF-based initiation mechanisms. These neural underpinnings may correspond to different challenges in terminating or initiating motor commands and underscore the nuanced neurobiological processes underlying speech disfluencies.

## Introduction

More than 50 million adults worldwide live with persistent developmental stuttering [1,2], which can have profound adverse effects on quality of life [3]. Stuttering is generally characterized by overt speech behaviors such as repetitions (i.e., repeated sounds or syllables), prolongations (i.e., extended duration of a sound), and blocks (i.e., temporary stoppage of airflow or vocalization). However, the prevalence of each type can vary widely across individuals. Prior research has highlighted individual differences and potential subgroups in the expression of disfluencies among adults and children who stutter [4–8]. This heterogeneity presents challenges in identifying the underlying neural mechanisms of stuttering [9–12]; neuroimaging studies have struggled to offer a unified understanding of the brain regions involved, possibly because key insights are obscured by grouping all individuals who stutter together despite differences in underlying etiologies. A clearer mechanistic understanding of how specific neural control systems contribute to individual stuttering patterns could inform the development of more targeted, individualized treatment strategies, potentially improving therapy outcomes for adults who stutter (***AWS***) [13–19].

### The role of feedback and feedforward control in stuttering

Over the past several decades, a substantial number of neuroimaging studies have expanded our understanding of the neural mechanisms underlying stuttering. Researchers have found associations between disfluency rates and functional and structural anomalies in left inferior frontal gyrus, bilateral temporal regions, right frontal regions, and speech motor networks involving basal ganglia, thalamus, and sensorimotor cortex (see [9,20–22] for reviews). Increased cerebellar activity has also been consistently observed across studies, with many researchers arguing that it reflects a compensatory mechanism for timing deficits in AWS [23–31].

Two mechanisms that have garnered increasing interest for understanding stuttering are feedback (***FB***) control (i.e., integrating information to monitor and correct for errors) and feedforward (***FF***) control (i.e., adjusting the system through learning to preemptively avoid producing errors) of speech production. These control processes have been associated with distinct brain networks, as detailed in the Directions into the Velocities of Articulators (DIVA) model [32] and Gradient Order DIVA (GODIVA) model [33], which are theoretical frameworks that offer comprehensive accounts of the neural mechanisms involved in speech motor control. The DIVA model proposes that speech production relies on a combination of FB and FF control processes, highlighting the role of the cerebellum in fine-tuning motor plans and the basal ganglia in regulating the initiation of speech. The GODIVA model builds upon DIVA by further specifying the gradient organization of motor planning, with a focus on how complex articulatory movements are organized hierarchically across different levels of speech production. Together, these models offer valuable insight into how disruptions in the FB and FF systems could contribute to disfluencies like stuttering, particularly in the coordination of speech movements and error correction.

Over the years, there has been an increased focus on FB and FF control as a means of understanding the behavioral and neural findings associated with stuttering. Several leading etiological theories have implicated FB control in the cause of stuttering, suggesting that difficulties detecting errors and making adjustments during speech can lead to disfluencies [34–36]. These theories are supported by evidence from behavioral and neuroimaging studies, which reveal differences in FB-based processing [37–45], such as greater reliance on auditory FB [46–48], and anomalies in planum temporale and middle temporal gyrus, areas thought to contribute to FB control [18,49–51]. Other theories have proposed that deficient FF control is the primary cause of stuttering, suggesting that difficulties planning and/or initiating movements are at the core of disfluencies [9]. Consistent with these theories, behavioral and neuroimaging studies have revealed differences in FF-based processing [9,34–36,38–40,43,45] and anomalies in brain regions hypothesized to contribute to FF control [50,52,53].

A common theme across this literature is that individuals who stutter are not a homogeneous group but rather exhibit diverse neural profiles [9,10,13,18]. Indeed, in many of the studies mentioned above, researchers found that only a subset of AWS relied more on auditory FB [48] or exhibited strengthened FB or FF network connectivity following treatment [50]. Researchers have also identified subgroups based on atypical symmetry of planum temporale [18], which is hypothesized to be associated with FB processing, and atypical caudate anatomy [52] and underactivity in basal ganglia pathways [9,13], both of which are hypothesized to be associated with FF processing. This within-population variability suggests that FB and FF control may influence stuttering to varying degrees across different individuals who stutter.

### Prior literature on neural underpinnings of specific disfluency types

Despite the heterogeneity in stuttering types and in the brain mechanisms involved in stuttering across AWS, no study to our knowledge has specifically examined the neural underpinnings of the three primary disfluency types. One prior study used pattern classification to automatically categorize disfluencies that are more typical (***MT***) of stuttering (i.e., part-word repetitions, prolongations, and broken words) and less typical (***LT***) of stuttering (i.e., incomplete phrases, revisions, interjections, and phrase repetitions) in AWS and neurotypical speakers using corresponding task-based fMRI data [54]. The authors found that left inferior frontal cortex and bilateral precuneus showed higher activity for MT disfluencies, while left putamen and right cerebellum showed higher activity for LT disfluencies. These results demonstrate that different clusters of disfluencies (i.e., MT versus LT) can be attributed to differences in brain activity, but they address the neural underpinnings of MT disfluencies as a whole rather than of the individual disfluency types.

While less direct than studies examining human behavior, computational modeling can be used to provide insights into the neural processes underlying speech. The DIVA and GODIVA models, specifically, have been used to model the impact of brain function hypothesized to be involved in FB and FF control. Over five decades ago, a series of seminal studies provided evidence proposing that repetitions may be related to FB control, specifically; these studies demonstrated an absence/reduction of repetitions when auditory FB was not available due to masking [55–58]. Based on this work, DIVA model simulations tested the association between FB control and repetitions and demonstrated that overreliance on FB control (and thus oversensitivity to speech errors) could lead to repeated or prolonged phonemes [59]. By increasing the gain on the FB control system, the simulations induced repetition errors that were acoustically and kinematically similar to those of AWS in prior behavioral studies [60–64]. In another simulation, masking noise was added to the auditory FB signal and successfully reduced repetitions, indicating that noise prevents detection (and subsequent overcorrection) of errors, consistent with the aforementioned behavioral studies in AWS [65,66].

Neural modeling has also provided evidence of the potential contribution of FF control to the production of blocks. GODIVA simulations investigated the hypothesis that elevated dopamine levels in a basal ganglia thalamo-cortical (BGTC) circuit could lead to a failure to initiate the upcoming syllable in a timely manner, suggesting that blocks could arise from impairments in FF control [67,68]. The authors mimicked the effects of excess dopamine in the system by artificially reducing the signal to noise ratio between the motor program for the upcoming syllable and those for the other syllables. This manipulation resulted in the model’s inability to bias the upcoming syllable for selection, ultimately causing a block in speech production. These findings underscore the possible role of the FF control network in the timely initiation of speech sounds.

### The current study

The simulation experiments discussed above illustrate that targeted disruptions to the FB and FF networks for speech can lead to distinct stuttering behaviors [59,67,68]. This insight motivated the current study, which investigates how feedforward (FF) and feedback (FB) control networks relate to different stuttering types by examining correlations between disfluency patterns and resting-state connectivity in individuals with persistent stuttering. While the aforementioned studies primarily focused on task-based fMRI to examine neural mechanisms underlying speech production, resting-state functional connectivity offers complementary insights by capturing a stable measure of functional network organization, reflecting the brain’s inherent activity patterns and neural traits that persist across contexts. This approach allows us to investigate baseline connectivity patterns that may underlie susceptibility to disfluencies, rather than neural responses constrained by specific task performance. Furthermore, resting-state functional connectivity analysis circumvents challenges commonly encountered in task-based studies of stuttering, such as difficulty in eliciting spontaneous disfluencies under controlled experimental conditions and increased motion artifacts during speech production tasks. Prior work has also demonstrated strong correlations between resting state functional connectivity and specific behaviors (e.g., inhibitory control in attention-deficit/hyperactivity disorder) [69] and its ability to distinguish subtypes of social-cognitive and neurocognitive differences in clinical populations [70,71].

Based on the aforementioned literature [55–59,67,68], we hypothesize that repetitions and prolongations will be primarily associated with *increased* connectivity in the FB control network, reflecting an overreliance on FB mechanisms, while blocks will be primarily associated with *reduced* connectivity in the FF control network, reflecting weakened FF mechanisms. Notably, although the DIVA model does not explicitly define this distinction, we divided Cb into anterior and posterior regions to reflect functional differences; anterior regions are primarily involved in motor aspects of speech, while posterior regions are associated with higher-level cognitive functions [72,73].

Our goal is to explore potential associations that could guide future research into more targeted and individualized models of stuttering. For example, by identifying how specific neural control systems contribute to different disfluency types, we may eventually be able to tailor therapies that target the dominant mechanisms affecting each individual. However, we do not aim to make definitive conclusions at this stage, but rather to identify trends that might warrant further investigation in subsequent studies. Finally, while in this study we focused on overt speech disruptions, we acknowledge that these behaviors do not capture the full complexity of the stuttering experience. However, focusing on these observable features provides a quantifiable and reproducible starting point for identifying potential neural mechanisms, which can later be expanded to encompass the broader phenomenology of stuttering.

## Materials and methods

### Participants

Participants included 20 AWS (15 male, 5 female; aged 18−52 years, mean age = 30.50 + /- 11.89 years). All participants were diagnosed with stuttering based on self-identification as a person who stutters during a screening interview and self-report that certain sounds and social contexts impact their overt symptoms. The ratio of males to females in this study is consistent with the prevalence of persistent developmental stuttering in the national population [74]. All participants were native English speakers, reported normal hearing and vision, had no prior history of speech, language, or hearing disorders other than stuttering, and self-identified as stuttering during a screening interview. Using the Edinburgh Handedness Inventory [75], all participants were found to be right-handed (i.e., scoring greater than 40). The study was approved by the Boston University Institutional Review Board (#2421) and all participants provided written informed consent. Data were accessed on November 9, 2023 for research purposes. The author conducting the analyses did not have access to information that could identify individual participants. See Table 1 for demographic information.

**Table 1:** Demographic information and stuttering behavior data of adults who stutter. Percentages are calculated by dividing number of repetitions, prolongations, or blocks by total number of syllables produced.

Participant ID	Age	Gender	Scanner	SSI-4 (range 9–42)	Total Number of Syllables	Repetition Raw Count and Percentage	Prolongation Raw Count and Percentage	Block Raw Count and Percentage
AWS01	19	F	MGH	28	791	11 (1.39%)	0 (0%)	54 (6.83%)
AWS02	22	F	MGH	31	542	18 (3.32%)	11 (2.03%)	84 (15.50%)
AWS03	31	F	MGH	10	704	3 (0.43%)	0 (0%)	9 (1.28%)
AWS04	31	F	MGH	30	737	6 (0.81%)	4 (0.54%)	45 (6.11%)
AWS05	18	F	BU	14	770	1 (0.13%)	0 (0%)	27 (3.51%)
AWS06	23	M	MGH	20	868	12 (1.38%)	5 (0.58%)	24 (2.77%)
AWS07	19	M	MGH	9	346	2 (0.58%)	0 (0%)	4 (1.16%)
AWS08	29	M	MGH	33	880	24 (2.73%)	1 (0.11%)	97 (11.02%)
AWS09	58	M	MGH	14	857	21 (2.45%)	1 (0.12%)	6 (0.70%)
AWS10	42	M	MGH	22	1069	21 (1.96%)	1 (0.09%)	13 (1.22%)
AWS11	22	M	MGH	42	248	4 (1.61%)	3 (1.21%)	24 (9.68%)
AWS12	52	M	MGH	27	698	10 (1.43%)	0 (0%)	29 (4.15%)
AWS13	44	M	BU	20	793	12 (1.51%)	4 (0.50%)	18 (2.27%)
AWS14	29	M	BU	14	847	16 (1.89%)	9 (1.06%)	15 (1.77%)
AWS15	20	M	BU	18	799	1 (0.12%)	6 (0.75%)	18 (2.25%)
AWS16	43	M	BU	27	913	49 (5.37%)	17 (1.86%)	15 (1.64%)
AWS17	35	M	BU	30	814	11 (1.35%)	8 (0.98%)	43 (5.28%)
AWS18	22	M	BU	27	759	10 (1.32%)	1 (0.13%)	28 (3.69%)
AWS19	21	M	BU	19	827	14 (1.69%)	7 (0.85%)	33 (3.99%)
AWS20	20	M	BU	24	812	1 (0.12%)	1 (0.12%)	33 (4.06%)

SSI-4 = Stuttering Severity Index–Fourth Edition; AWS = adults who stutter; F = female; M = male; MGH = Massachusetts General Hospital; BU = Boston University.

### Behavioral data acquisition

Stuttering severity and disfluency types (i.e., repetitions, prolongations, and blocks) were derived from the Stuttering Severity Instrument – Fourth Edition (SSI-4 [76];), which included three speech samples: an in-person conversation, a phone conversation, and the Grandfather reading passage [77]. The topic of the in-person conversational samples varied across participants, with examples such as their current job, hobbies, or future vacation plans, and they were 4–6 minutes and 415 syllables long, on average. For the reading sample, each participant was told to: “Please read this paragraph. Begin when you are ready.” Each reading sample was 2–3 minutes and 170 syllables long, on average. Lastly, the phone call samples were 2–3 minutes and 200 syllables long, on average. The frequency of each stuttering type was calculated as the average across all three speech samples, giving equal consideration to each task. All speech samples were audio and video recorded. For each sample, a licensed speech-language pathologist who was board certified in fluency calculated the number of total syllables produced, total stuttered syllables, and total number of each disfluency type.

Stuttering behaviors were operationally defined based on well-established criteria from the literature [78]. The three primary disfluency types—repetitions, prolongations, and blocks—were categorized according to standardized definitions: repetitions refer to the involuntary repetition of sounds, syllables, or words; prolongations refer to the extended duration of speech sounds; and blocks refer to interruptions in the flow of speech, often accompanied by physical tension. These behaviors were reliably identified and scored using established coding guidelines to ensure consistency across participants. When mixed stuttering was present, which involves the occurrence of two or more different stuttering types in a single stuttering moment (e.g., a repetition combined with a prolongation or block), the stutter was classified according to the dominant disfluency type within that occurrence. “Dominance” was operationalized as the feature occupying the greater proportion of the stuttering moment. In rare cases where no type was clearly dominant, the classification was determined based on the first disfluency to occur in the sequence. Because blocks are often perceptually salient, coders were trained to apply this rule conservatively to avoid over-classification of blocks when they occurred in combination with repetitions or prolongations. This approach ensured that mixed disfluencies were consistently classified while minimizing the risk that blocks would be disproportionately coded as dominant. To confirm reliability, inter-rater agreement between three separate raters (i.e., the original rater and two additional speech-language pathologists) was assessed on 20% of the data. This analysis demonstrated an ICC of.96 for repetitions,.80 for prolongations, and.76 for blocks, all of which are within the range of good to excellent reliability. See Table 1 for stuttering severity and stuttering type data.

### Resting state data acquisition

MRI data were acquired approximately two months, on average, after the behavioral data acquisition. Data were acquired at two sites: the Athinoula A. Martinos Center for Biomedical Imaging at the Massachusetts General Hospital (MGH) in Charlestown, MA and the Cognitive Neuroimaging Center at Boston University (BU). Images were acquired using a Siemens 3T MAGNETOM Skyra scanner with a 32-channel head coil at MGH and a Siemens 3T Prisma scanner with a 64-channel head coil at BU. To acquire resting state functional connectivity data, participants were positioned supine in the scanner for six minutes. They were given the following instructions: “Please lie quietly with your eyes open and observe the cross. During this scan you should let your mind wander freely. Please remain awake and keep still during this scan. This scan will last six minutes.” A gradient echo, echo-planar imaging BOLD sequence (repetition time [TR] = 1130 ms, echo time = 30 ms, flip angle = 60°, simultaneous multi-slice factor = 2) was used to collect 315 continuous volumes. Each functional volume comprised 51 axial slices (72 × 72 matrix) acquired in interleaved order (in-plane resolution = 2.97 x 2.97 mm^2^; slice thickness = 3.0 mm with no gap). A high-resolution T1-weighted whole-brain structural image was also collected from each participant to anatomically localize the functional data (MPRAGE [magnetization-prepared rapid gradient-echo] sequence; 256 × 256 × 176 mm^3^ volume, with a 1-mm isotropic resolution; TR = 2530 ms; echo time = 1.69 ms; flip angle = 7°).

### Functional connectivity data analysis

#### Preprocessing.

Preprocessing was conducted using the CONN toolbox [79], which is one of the most widely used tools for resting-state fMRI analyses, as it provides a robust framework for functional connectivity analyses and has been validated in the field [80,81]. Two distinct pipelines were employed: a surface-based approach to extract cortical activation and a volume-based approach to extract subcortical and cerebellar activation. Our decision to combine surface-based methods for cortical regions and volumetric approaches for subcortical areas is guided by previous work demonstrating that surface-based analysis provides superior alignment of cortical folding patterns [82–84]. In contrast, volumetric approaches are recommended for subcortical structures, where complex three-dimensional architecture cannot be effectively captured by surface-based methods [85]. Using both approaches leverages the unique strengths of each method, maximizing anatomical accuracy across all relevant brain regions.

Preprocessing common to both pipelines included slice time correction, motion correction, and outlier detection, followed by denoising with aCompCor [86]. Using Statistical Parametric Mapping (SPM12)’s realign and unwarp procedure, functional images were adjusted for any misalignments based on the mean subject image and corrected for motion-by-inhomogeneity interactions [87]. Artifact detection tools (https://www.nitrc.org/projects/artifact_ detect/) identified outlier scans based on motion displacement (scan-to-scan motion threshold of 0.9 mm) and mean signal change (scan-to-scan signal change threshold of 5 SDs above the mean). To remove any extraneous motion, physiological, and artifactual effects from the BOLD signal for each subject, denoising was performed using a linear regression model in each seed ROI and every voxel in the smoothed brain volume, including five white matter regressors, five cerebrospinal fluid regressors, six subject motion parameters, and scrubbing regressors to exclude the effects of outlier scans, as well as individual regressors for each run and session, task condition, and error trial [88].

For surface-based analyses, FreeSurfer [83] was employed to preprocess the T1 structural volumes by removing non-brain components [89] and segmenting the brain into gray matter, white matter, and cerebrospinal fluid components [90]. Cortical surface reconstructions for each hemisphere were then generated [91] and registered to a standard surface template (*fsaverage*). The resting state fMRI volume was co-registered to the structural volume to align the functional and anatomical data. Blood oxygen level dependent (BOLD) responses from the resting state fMRI data were then mapped to their corresponding vertices on the cortical surface. The surface-based representation of the resting state fMRI data was smoothed using iterative diffusion smoothing with 40 diffusion steps (equivalent to an 8-mm full-width half-maximum smoothing kernel; [92]) for vertex-level analyses.

For volume-based analyses, functional volumes were co-registered to the T1 structural images and then segmented and normalized to Montréal Neurological Institute (MNI) space after outlier detection using SPM12 [93]. The structural T1 images were centered, segmented, and normalized with SPM12 to ensure accurate alignment with the functional data. Following co-registration, volumes were smoothed with an 8-mm full-width half-maximum smoothing kernel for voxel-level analyses. Afterward, a mask was applied to include only voxels within subcortical structures for subsequent analyses. After preprocessing, two AWS participants were excluded from further analyses (not included in the *Subjects* section): one due to excessive head motion in the scanner (> 1.5 mm average scan-to-scan motion) and the other due to structural brain abnormalities.

#### Region-of-interest definition.

Cortical regions-of-interest (***ROIs***) were identified using a modified version of the SpeechLab atlas, as described in Cai and colleagues (2014). This atlas divides the cortex into macro-anatomically defined ROIs designed specifically for speech studies. The labeling was performed by mapping the atlas from the FreeSurfer *fsaverage* cortical surface template onto each individual’s surface reconstruction.

Subcortical and cerebellar ROIs were sourced from multiple different atlases. Thalamic ROIs were obtained from the mean atlas of thalamic nuclei [94]. Basal ganglia ROIs were extracted from the nonlinear normalized probabilistic atlas of basal ganglia [95]. Each ROI was thresholded at a minimum probability of 33% and combined into a single labeled volume within the atlas’s native space (the MNI104 template). Cerebellar ROIs were extracted from the cerebellar networks included in the CONN toolbox. Each atlas was nonlinearly registered to the SPM12 MNI152 template and then merged into a single labeled volume.

#### Region-of-interest-based connectivity analyses.

ROI-based connectivity analyses were performed using the CONN toolbox, which computed the Fisher-transformed temporal correlation between ROI pairs. ROI-to-ROI correlations were then extracted for connections described in the DIVA and GODIVA models’ FB and FF control networks (including anterior and posterior Cb). Our focus on DIVA-based connections was a strategic choice to: (1) maximize the power of the analysis given our small sample size, and (2) directly test hypotheses derived from the model, ensuring the analysis was grounded in established theoretical and empirical research. By concentrating on a targeted set of connections directly relevant to the speech production process, we aimed to reduce the risk of Type II errors and maintain sufficient sensitivity to detect meaningful effects. To reduce the total number of connections analyzed, we focused on left-lateralized connections and eliminated right hemisphere connections that were functionally redundant with those in the left, retaining only select right hemisphere connections (i.e., connections to right ventral premotor cortex and right cerebellum). In light of findings of increased right hemisphere involvement in stuttering, we also included connections with right brain regions that have been consistently found to be active in prior literature in AWS (see 9,20,22 for reviews). In the end, a total of 58 Fisher-transformed correlation coefficients were included as predictors in the subsequent statistical analyses.

### Statistical analyses

Statistical analyses were performed using R. To identify the connections that most strongly predicted each disfluency type, we created 1000 bootstrapped samples through random sampling with replacement from the original dataset, generating multiple resampled datasets to assess the stability of our estimates. Both the predictors (i.e., the DIVA/GODIVA connections) and the response variables (i.e., repetitions, prolongations, and blocks) were bootstrapped, ensuring a comprehensive evaluation of the robustness of our model. Bootstrapping was applied to mitigate potential overfitting and provide more reliable estimates of the model coefficients [96]. This approach allowed us to assess the stability of the relationships between predictors and response variables, improving the generalizability and confidence in our findings.

For each bootstrapped sample, we conducted three Least Absolute Shrinkage and Selection Operator (LASSO) regressions—one for each disfluency type—using the *cv.glmnet* function from the *glmnet* package. The predictors included the 58 aforementioned Fisher-transformed correlation coefficients, while the outcome was percentage of each disfluency type across all syllables in the speech sample. To control for potential scanner-related effects, the scanner variable was included in each model, with binary encoding (1 for MGH and 0 for BU). Statistical control of scanner effects accounts for differences in average connectivity due to acquisition parameter variability across sites. We also conducted Levene’s tests to assess potential heteroscedasticity introduced by inter-site differences in variance. The results did not indicate a significant effect (see Table S1 in Supplementary Material), suggesting that further normalization across sites was not necessary. The LASSO models were then fitted with the optimal lambda (regularization parameter) determined via cross-validation. LASSO regression prevents overfitting when working with small, high-dimensionality datasets, and is one of the most commonly used penalized regression methods [97].

Next, we calculated the average coefficient values and standard deviation across the 1000 iterations for each predictor. To assess whether each coefficient value was statistically significantly different from zero, we constructed 95% confidence intervals (CIs) for each raw LASSO coefficient using the standard errors derived from the bootstrapped samples. A predictor was considered statistically significant if its 95% CI did not include zero, indicating a consistent, non-random effect across the resampled datasets. To account for multiple comparisons, we applied a Bonferroni correction, adjusting the threshold for statistical significance. This bootstrapped CI approach provides a robust measure of significance, particularly well-suited for high-dimensional data, as it accounts for variability in the estimates without relying on strict parametric assumptions.

To ensure the robustness of our coefficient estimates, we applied a stability selection method [98], which involved calculating the frequency of non-zero coefficients for each predictor across the 1000 iterations. This method helped to identify which predictors were consistently influential. To determine a cutoff for variable inclusion based on stability selection frequencies, we used a data-driven heuristic inspired by scree plot and elbow methods. Specifically, we identified the index at which the first prominent drop in selection frequency occurred among predictors, ranked by their selection count across bootstrap samples. This point was interpreted as the last relatively stable variable before the onset of a sharp decline, indicating a transition from highly stable to less consistently selected predictors. Although this approach does not provide formal control of false discoveries, it offers a pragmatic balance between sparsity and reproducibility in the context of exploratory feature selection. Compared to traditional elbow methods, our “first drop” heuristic imposes a more conservative criterion for feature selection. Rather than identifying the point of diminishing returns, it targets the onset of instability in selection frequencies, yielding a smaller but more stable set of predictors. This stricter threshold prioritizes reproducibility over model complexity, aligning with our goal of identifying robust, high-confidence variables.

## Results

### Behavioral analysis

In our study of 20 participants, the mean SSI-4 score was 22.95 (SD = 8.48), indicating mild to moderate severity across the sample. On average, repetitions made up 1.21% (SD = 1.14) of all syllables spoken, prolongations made up 0.47% (SD = 0.57), and blocks made up 3.72% (SD = 4.47). Repetitions and prolongations comprised a relatively small proportion of the syllables spoken, but there was considerable variability among participants, with some showing minimal to no repetitions/prolongations and others displaying more frequent occurrences. Blocks were more common, making up a larger proportion of the total syllables produced compared to repetitions and prolongations. However, the large standard deviation also suggests considerable variability in the frequency of blocks.

### Brain-behavior analysis

**Fig 1:**
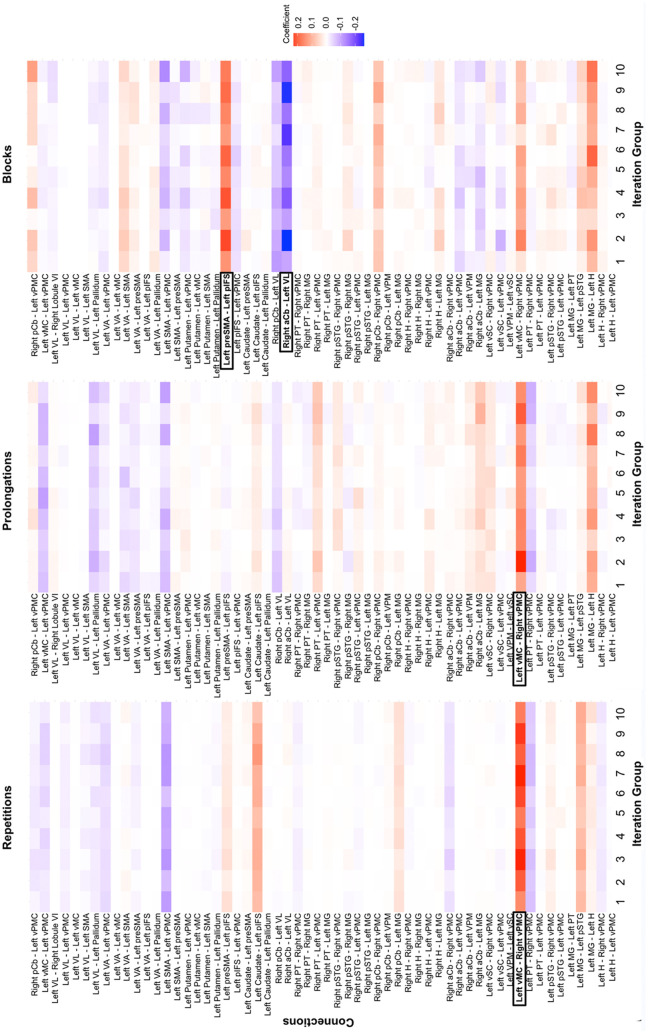
Heatmap of LASSO regression coefficients averaged across 1000 bootstrapped iterations. Color intensity = coefficient magnitude; black rectangles = most consistently selected predictors; a/pCb = anterior/posterior cerebellum; GP = globus pallidus; H = Heschl’s gyrus; MG = medial geniculate thalamic nucleus; pAC = posterior auditory cortex; pIFS = posterior inferior frontal sulcus; preSMA = presupplementary motor area; pSTG = posterior superior temporal cortex; PT = planum temporale; SMA = supplementary motor area; VA = ventral anterior thalamic nucleus; VL = ventral lateral thalamic nucleus; vMC = ventral motor cortex; vPMC = ventral premotor cortex; VPM = ventral posterior medial thalamic nucleus; vSC = ventral somatosensory cortex.

The LASSO regression analysis aimed to identify resting state functional connectivity patterns underlying different disfluency types. After conducting bootstrapping with 1000 resampled datasets, LASSO regressions were performed for each disfluency type. Optimal lambda values, which control the strength of the regularization, were determined through cross-validation, minimizing the mean squared error. These optimal lambda values were then used to fit the LASSO models, resulting in a mean coefficient value (also referred to as “estimate” below) across the 1000 iterations for each regression. For repetitions, prolongations, and blocks, 27, 26, and 23 predictors, respectively, showed statistically significant effects. In each case, the confidence intervals did not include zero, indicating consistent and non-random relationships across the resampled datasets (see Table S2 in Supplementary Material). Notably, although blocks were associated with fewer overall predictors than repetitions or prolongations, the predictors that did emerge for blocks showed larger effect estimates and required greater regularization, suggesting stronger and potentially more robust brain–behavior relationships. By contrast, prolongations exhibited fewer and weaker associations, consistent with their relatively lower prevalence in the behavioral data. These results, derived from optimal lambda values determined via cross-validation, demonstrate robust and significant functional connectivity patterns related to disfluency types. See Fig 1 for a heatmap of LASSO regression coefficients from the most optimal model across the bootstrapped iterations.

**Fig 2:**
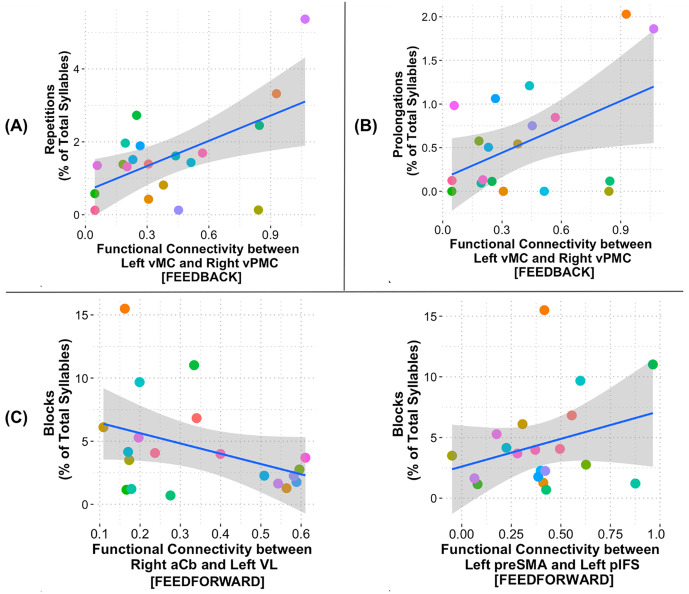
Correlations between repetition rate (panel A), prolongation rate (panel B), and block rate (panel C) and functional connectivity in the feedback and feedforward control networks. Colored dots = individual participants; aCb = anterior cerebellum; pIFS = posterior inferior frontal sulcus; preSMA = presupplementary motor area; VL = ventral lateral thalamic nucleus; vMC = ventral motor cortex; vPMC = ventral premotor cortex.

The stability selection method was then conducted to identify the most consistently influential predictors across the 1000 iterations. Lastly, a data-driven cutoff was identified based on the point just before the largest drop in selection frequency, capturing the most consistently selected predictors across model iterations (see Fig S1 in Supplementary Material). The following connections were the most consistent across iterations (outlined in black rectangles in Fig 1): Repetitions (average optimal λ = 0.09) were primarily predicted by increased connectivity between *left ventral motor cortex (vMC)* and *right ventral premotor cortex (vPMC)* (FB control network; estimate = 0.21, SE = 0.008); prolongations (average optimal λ = 0.11) were primarily predicted by increased connectivity between *left ventral motor cortex (vMC)* and *right ventral premotor cortex (vPMC)* (FB control network; estimate = 0.04, SE = 0.003); and blocks (average optimal λ = 0.75) were primarily predicted by reduced connectivity between *right anterior cerebellum (aCb)* and *left ventral lateral thalamic nucleus (VL)* (FF control network; estimate = −0.18, SE = 0.015) and increased connectivity between *left presupplementary motor cortex (preSMA)* and *left posterior inferior frontal sulcus (pIFS)* (FF control network; estimate = 0.17, SE = 0.014). See Fig 2 for the correlation plots of each disfluency type and area of functional connectivity.

Lambda values indicate the degree of regularization applied in the LASSO regression models. Lower lambda values for repetitions and prolongations suggest that these types of disfluencies are more sensitive to subtle variations in connectivity. Conversely, a higher lambda for blocks implies that more regularization was needed, indicating potentially greater complexity or variability in the predictors. For repetitions, the relatively higher estimate (0.21) with a lower standard error (0.008) for the left vMC to right vPMC connection suggests a strong association. For prolongations, the lower estimate (0.04) with a small standard error (0.003) for the vMC to vPMC connection indicates weaker (though still significant) association compared to repetitions. For blocks, the negative estimate (−0.18) with a relatively higher standard error (0.015) for the aCb to VL connection suggests a stronger but more variable association, while the positive estimate (0.17) with a similar standard error (0.014) for the preSMA to pIFS connection indicates a strong and consistent association. Overall, the varying lambda values reflect the different levels of complexity and regularization needed for each type of disfluency. The estimates provide insight into which connectivity patterns are most strongly associated with each disfluency type, with stronger associations seen in repetitions and blocks compared to prolongations.

## Discussion

**Fig 3:**
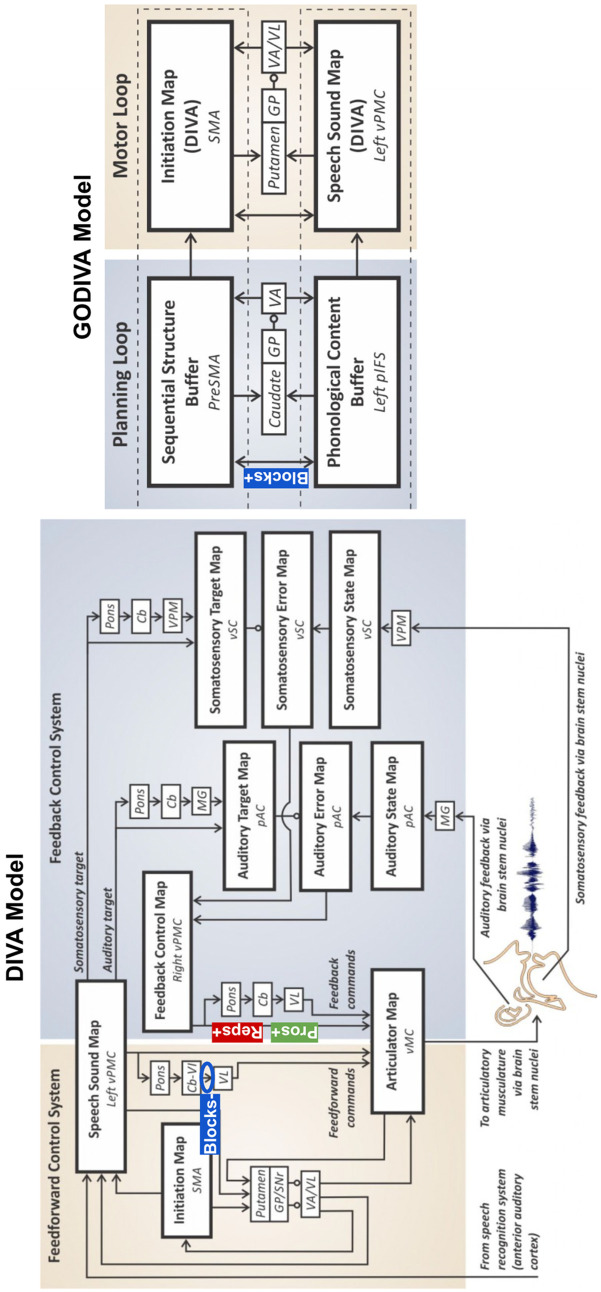
Current findings within the frameworks of Directions Into Velocities of Articulators (DIVA) and Gradient Order DIVA (GODIVA). Cb = cerebellum; GP = globus pallidus; MG = medial geniculate thalamic nucleus; pAC = posterior auditory cortex; pIFS = posterior inferior frontal sulcus; SMA = supplementary motor area; VA = ventral anterior thalamic nucleus; VL = ventral lateral thalamic nucleus; vMC = ventral motor cortex; VPM = ventral posterior medial thalamic nucleus; vPMC = ventral primary motor cortex; vSC = ventral somatosensory cortex; + = positive correlation between disfluency rate and connection strength; - = negative correlation between disfluency rate and connection strength; pros = prolongations; reps = repetitions.

**Fig 4:**
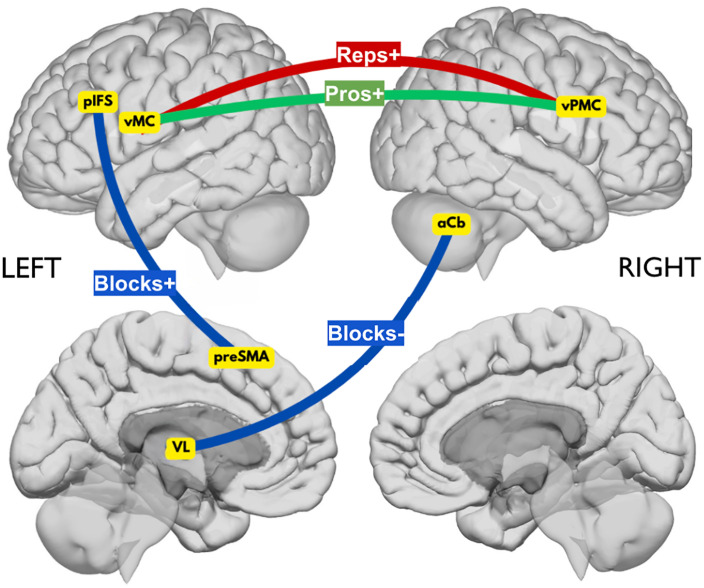
Schematic of the brain connections associated with each disfluency type. Red line = repetitions; green line = prolongations; blue line = blocks; aCb = anterior cerebellum; pIFS = posterior inferior frontal sulcus; preSMA = presupplementary motor area; VL = ventral lateral thalamic nucleus; vMC = ventral motor cortex; vPMC = ventral premotor cortex; + = positive correlation between disfluency rate and connection strength; - = negative correlation between disfluency rate and connection strength; pros = prolongations; reps = repetitions.

The present study investigated the underlying neural mechanisms of different stuttering-like disfluency types (i.e., repetitions, prolongations, and blocks) using resting state functional connectivity data and a LASSO regression analysis. While we hypothesized that repetitions and prolongations may reflect greater reliance on FB-based corrective mechanisms and that blocks may stem from disruptions in FF-based initiation mechanisms, our findings suggest only partially distinct connectivity patterns across disfluency types. Rather than implying entirely separate neural pathways, the results point to overlapping yet differentially weighted contributions of FB and FF systems, which may help explain the diversity of symptom expression in AWS. See Fig 3 for the findings within the frameworks of the DIVA and GODIVA models and Fig 4 for a schematic of the brain connections associated with each disfluency type.

### Repetitions and prolongations may be associated with increased reliance on FB-based corrective mechanisms

Repetitions and prolongations were positively associated with increased connectivity between left vMC and right vPMC. This finding is in line with the prior literature suggesting a greater reliance on auditory FB for AWS [46–48] and DIVA simulations demonstrating a possible overreliance on FB control that causes the system to “reset” and repeat the motor program [59]. According to the DIVA model, a FB Control Map in right vPMC transforms sensory errors (the difference between the intended speech sound and the current sensory state) into corrective motor commands; an Articulator Map in left (and right) vMC is responsible for executing the motor commands. Therefore, increased connectivity between these regions may reflect increased reliance on FB-based corrective mechanisms. This increased reliance may interfere with the initiation of FF-based termination commands or could be a response to already disrupted timing signals in the FF control system. In either interpretation, the heightened dependence on FB-based corrective mechanisms could lead to repeated or prolonged sounds as the system continues to adjust and correct the output instead of efficiently transitioning to the next motor command. Often referred to as a hypervigilant FB control system, this account is consistent with Chang and colleagues (2019) review of the functional and neuroanatomical bases of developmental stuttering. The authors conclude that within the context of the DIVA/GODIVA framework, stuttering results from an impairment in the FF control system in the left hemisphere, which, in turn, leads to an overreliance on the FB control system in the right hemisphere [99]. More recently, in a longitudinal task-based fMRI study examining spontaneous speech in children who stutter, Chow and colleagues (2023) found that stuttering was linked to a functional anomaly in left vPMC, which extends to right premotor cortex with age. The authors concluded that right-hemisphere hyperactivation in AWS may develop later in life and, therefore, reflect unsuccessful or maladaptive compensatory behavior rather than a causal mechanism of stuttering [100].

### Blocks may be associated with disrupted subcortical timing mechanisms and continued reliance on FF-based cortical timing mechanisms

In contrast to repetitions and prolongations, blocks were negatively associated with connectivity between right aCb and left VL. This finding is consistent with the growing body of literature supporting a compensatory role of Cb in stuttering [23–31]. In the FF control system in the DIVA model, there is a direct cortico-cortical pathway (i.e., left vPMC to left vMC) and an indirect pathway that involve the right aCb to left VL connection. Based on prior work suggesting that Cb contains sensory representations that influence corrective movements [101–103], the DIVA model proposes that right Cb contributes to FF motor commands, likely through left VL, which is the main source of cerebellar input to motor cortex.

The right aCb to left VL connection is thought to play a key role in refining and optimizing motor signals prior to their initiation. While much of the supporting literature has focused on the cerebellum’s role in manual motor coordination [101–103], there is growing evidence that these same predictive control mechanisms generalize to speech motor control. Similar to limb control, speech movements rely on the cerebellum to generate internal models that anticipate the sensory consequences of motor commands, allowing for the smooth and timely execution of complex motor sequences [104]. In speech, this function is particularly critical given the rapid and precise coordination required across multiple articulators. Furthermore, neuroimaging studies have shown overlapping cerebellar regions involved in both limb and speech tasks, suggesting shared computational principles [105,106]. Therefore, disruptions in cerebellar-thalamic connectivity, such as reduced connectivity between the right aCb and left VL, may reflect impairments in these predictive mechanisms, leading to difficulties initiating or coordinating speech movements. This may help explain our finding that reduced connectivity in this FF circuit is associated with an increased frequency of blocks. Of note, blocks also showed the strongest and most robust associations overall compared to repetitions and prolongations, consistent with their greater behavioral prevalence in our sample. This pattern suggests that blocks may be more tightly coupled to identifiable neural circuitry, whereas repetitions and especially prolongations may reflect more subtle or variable connectivity differences.

Blocks were also positively associated with connectivity between left pIFS and left preSMA, which are connected via the frontal aslant tract [107]. Given that preSMA projects to and is influenced by different regions of the basal ganglia and is a critical component of the BGTC motor network [108], our findings are consistent with those of Civier and colleagues in suggesting that abnormalities in the BGTC motor network may lead to initiation difficulties associated with stuttering [67,68]. This finding also aligns with prior resting state functional connectivity studies, which demonstrated the involvement of left inferior frontal gyrus [109] and, specifically, left pIFS [110] in stuttering.

According to the GODIVA model, a Phonological Content Buffer in left pIFS stores phonological representations of intended speech sounds, while a Sequential Structure Buffer in left preSMA organizes this content into a coherent and timed sequence. When acquiring a new speech sequence, speakers initially depend on cortical structures, such as preSMA and pIFS, to guide the sequencing and initiation of sounds [53]. As learning progresses, there is a shift towards greater reliance on subcortical structures, such as basal ganglia, thalamus, and Cb [53]. Our finding of decreased connectivity between aCb and VL suggests that these subcortical structures may not fully engage in learning or fine-tuning motor commands. The failure of Cb to integrate and optimize these commands may lead to an increased dependency on cortical mechanisms. The observed association between increased connectivity between preSMA and pIFS and increased blocks may, therefore, indicate a continued reliance on cortical areas for the sequencing and initiation of sounds, reflecting a suboptimal transition to subcortical control. Thus, AWS who have more blocks due to reduced subcortical connectivity may attempt to compensate (unsuccessfully) using cortical connections. A recent structural imaging study by Neef and colleagues (2018) also found a positive association between motor signs of stuttering and frontal aslant tract connectivity strength. The authors noted that both structures coordinate with subthalamic nucleus to mediate fast global inhibition, and an overly active global response suppression mechanism could underlie the ability to smoothly execute motor actions, which may manifest as stuttering [111]. Interestingly, pIFS has also been implicated in apraxia of speech [53,112,113]—a condition that primarily affects the ability to plan and sequence the movements necessary for speech, which can manifest as blocks or prolonged pauses.

### Integrating perspectives beyond DIVA and GODIVA

While we interpreted our findings within the DIVA and GODIVA frameworks, which emphasize the role of sensorimotor integration and control mechanisms in speech production, we acknowledge that other theoretical perspectives propose alternative mechanistic drivers of specific stuttering behaviors. For example, researchers have proposed that stuttering may evolve over the lifespan, progressing from relatively effortless repetitions to more tense, rapid, or irregular repetitions, prolongations, and eventually blocks [78]. This developmental trajectory is thought to reflect increasing awareness of, and reactions to, stuttering, as well as attempts to exert control over speech, which may engage different neural substrates than those implicated by DIVA and GODIVA models. Evidence from developmental studies of stuttering further supports this view, showing age-related shifts in disfluency patterns, with older individuals tending to exhibit more blocks relative to repetitions [6].

These contrasting perspectives highlight the possibility that different stuttering behaviors—and their neural correlates—may not be fully explained by a single model. Indeed, while DIVA and GODIVA primarily focus on disruptions in FB and FF control circuits, other models emphasize emotional and cognitive responses to stuttering. For example, the Covert Repair Hypothesis [114] attributes disfluencies to internal monitoring and error correction in speech planning, while the Dual Diathesis-Stress Model [115] emphasizes how emotional reactivity, self-regulation, and cognitive load shape stuttering expression. These alternative perspectives suggest additional involvement of networks related to self-monitoring, emotional regulation, and executive control, complementing sensorimotor accounts and underscoring the multifactorial nature of stuttering. As we continue to search for the cause of stuttering, it is essential to explore how these complementary mechanisms interact, potentially integrating neural signatures associated with both sensorimotor control and higher-order cognitive-emotional processes to provide a more comprehensive account of stuttering development and variability.

### Distinct versus common underlying causes of disfluency types

Stuttering is a multifaceted disorder with variable manifestations across individuals. While this study explored the hypothesis that repetitions, prolongations, and blocks may be differentially associated with FB and FF control systems, our findings do not suggest clean distinctions between the connectivity patterns supporting these systems. Rather, the results point to considerable overlap in the neural pathways associated with different disfluency types, indicating that these control mechanisms likely interact in complex and non-exclusive ways.

Notably, it is rare for individuals to exhibit only one type of disfluency; most present with a combination of repetitions, prolongations, and blocks. This behavioral overlap is reflected in the shared involvement of several ROI connections across disfluency types, suggesting that stuttering behaviors may arise from a common set of neural mechanisms modulated by individual differences in neural dynamics or control system balance. Instead of representing entirely distinct processes, repetitions, prolongations, and blocks may reflect different manifestations of disruptions within a shared speech motor network.

Thus, this study does not argue for entirely separate neural origins of disfluency types, but rather offers an initial step toward identifying network-level variations that may help explain individual differences in stuttering expression. Future work with larger samples, symptom-specific subgrouping, and inclusion of developmental cohorts may help clarify the extent to which certain neural pathways confer vulnerability to particular disfluency profiles, and how these relationships may shift with age or developmental stage.

### Limitations

While our study provides important insights into the neural mechanisms underlying distinct speech disfluency types, it has several limitations. First, the study involved a relatively small sample size (N = 20), which may limit statistical power. In addition, the presence of zero values for each disfluency type may have influenced correlation estimates. Thus, replication with a larger, more diverse cohort would enhance the generalizability and robustness of our findings.

Second, while we limited our investigation to DIVA- and GODIVA-based connections, we acknowledge the importance of demonstrating the specificity of our findings. Follow-up studies with larger sample sizes should incorporate random or control connections to directly compare the strength and relevance of the identified connections, helping to justify the a priori selection and further substantiate the findings’ specificity.

Third, our study did not account for secondary behaviors, which are often shaped by cognitive, emotional, and social factors. Incorporating measures of secondary behaviors, such as their frequency and timing relative to primary stuttering events, could help disentangle the extent to which neural activity associated with disfluencies may also reflect secondary motor responses. Additionally, studies utilizing multimodal neuroimaging and behavioral analyses could provide further insight into the interplay between motor planning disruptions and compensatory mechanisms in stuttering.

Fourth, while we examined disfluency patterns across different communication contexts, we did not model the potential influence of sample length or task duration on disfluency frequency. For example, in-person conversational samples tended to be longer than phone or reading samples, which may have increased opportunities for disfluencies to occur. Future analyses could include sample length as a covariate to better isolate effects related to context versus duration.

Fifth, although repetitions and prolongations were analyzed alongside blocks, these disfluency types made up a relatively small proportion of the total syllables produced. Given the reported variability in disfluency patterns across participants, this imbalance may limit the interpretability of neural associations specific to those less frequent disfluency types. Future studies with larger sample sizes and more balanced disfluency distributions could better support comparisons across disfluency types.

Sixth, classification of disfluency type introduces its own limitations, particularly when mixed or clustered disfluencies occur within the same moment. Although we coded by dominant disfluency type, blocks may have been more perceptible and thus more likely to be classified as dominant, potentially underestimating the prevalence of repetitions or prolongations when they co-occurred. This perceptual weighting could influence both frequency estimates and their associated neural patterns. Moreover, debates remain about the overall utility of disfluency-type classification. Some scholars have questioned whether such measures meaningfully advance our understanding or treatment of stuttering [116,117]. These critiques underscore the need for caution in interpreting findings tied to disfluency categories and illustrate the importance of developing complementary analytic approaches (e.g., multidimensional or continuous measures of stuttering severity).

Finally, our use of resting state functional connectivity limits the ability to capture real-time neural dynamics during moments of stuttering. As such, other factors may have influenced the frequency and type of disfluencies observed that were not accounted for in our analysis. These could include individual differences in cognitive-linguistic demands, speech context, and compensatory strategies developed over time. Moreover, internal states such as anxiety, stress, attention, and linguistic proficiency likely contribute to stuttering behaviors and may modulate neural network activity in ways not captured by resting-state measures. Future studies would benefit from incorporating direct assessments of these individual factors to better contextualize the neural mechanisms associated with disfluency patterns and improve the interpretability of resting-state connectivity findings. Additionally, task-based fMRI (e.g., controlled elicitation of stuttering behaviors; [118]), real-time neural recordings during speech, and comprehensive behavioral assessments could help disentangle these complex interactions. Longitudinal and task-based designs may further clarify the dynamic relationships between neural connectivity and situational influences, ultimately providing a more comprehensive understanding of the mechanisms underlying speech disfluencies.

### Theoretical and clinical implications

Overall, our findings provide evidence of partially distinct yet overlapping mechanisms underlying different stuttering behaviors, which has important theoretical implications for models of speech production and motor control. Specifically, these results support frameworks that differentiate between FB- and FF-based speech regulation, while also highlighting shared neural pathways. This view challenges the notion of a singular neural deficit in stuttering and instead points to a more nuanced, mechanism-specific perspective. Additionally, these findings motivate the need for theoretical models to account for individual variability in neural control strategies, which may inform more personalized approaches to understanding and treating stuttering.

From a clinical standpoint, further research in this area has the potential to inform the development of more targeted behavioral and medical interventions for individuals who stutter, ultimately improving treatment efficacy and personalization. For example, if repetitions and prolongations are associated with an overreliance on auditory FB mechanisms, behavioral strategies targeting enhanced control of auditory feedback, such as those using speech feedback manipulation (e.g., delayed auditory feedback), could benefit individuals exhibiting these disfluency types. Similarly, if blocks are associated with difficulties in initiating speech due to disruptions in the FF system and subcortical timing mechanisms, treatment strategies focusing on enhanced speech initiation or smoother transitions between speech motor programs could be more effective for individuals who primarily exhibit blocks.

Additional research could also inform future medical interventions (e.g., neuromodulation or pharmacological treatments) targeting specific neural pathways. Prior studies have reported increased fluency following stimulation to speech motor areas, including inferior frontal gyrus and supplementary motor area [119,120]. Similarly, pharmacological interventions have targeted dopaminergic pathways, given the proposed role of dopamine dysregulation in stuttering [121,122]. Building on these findings, individuals with FB-based disfluencies (e.g., repetitions, prolongations) may benefit from neuromodulation to the left vMC to right vPMC pathway, with the goal of recalibrating an overactive feedback system. For individuals with blocks, which may be linked to timing disruptions in subcortical circuits, pharmacological treatments targeting the basal ganglia or cerebellum could offer more tailored approaches to restore motor initiation and timing function.

In conclusion, these findings underscore the potential for developing personalized, targeted interventions that consider the different neural mechanisms involved in various stuttering behaviors. By targeting the underlying neural mechanisms associated with different disfluency types, both behavioral and medical treatments for stuttering could be made more effective and personalized. Further research with larger sample sizes will provide stronger evidence to guide the development of future behavioral and medical interventions, helping to identify the most effective targets for treatment based on specific neural pathways associated with different disfluency types.

## Supporting information

Table S1 Results of Levene’s tests assessing potential heteroscedasticity due to inter-site variance. (DOCX)

Table S2 Coefficient estimates, standard errors, and 95% confidence intervals of connections with statistically significant effects identified by each LASSO regression. (DOCX)

Fig S1 Selection frequencies plotted in decreasing order. Red point = cutoff used to distinguish stable from unstable predictors, identified as the last variable before the largest drop in selection frequency; aCb = anterior cerebellum; pIFS = posterior inferior frontal sulcus; preSMA = presupplementary motor area; VL = ventral lateral thalamic nucleus; vMC = ventral motor cortex; vPMC = ventral premotor cortex.
(TIF)

## References

[1] Smith A. Stuttering: a unified approach to a multifactorial, dynamic disorder. In: Bernstein Ratner N, Healey EC, editors. Stuttering research and practice: bridging the gap. 1st ed. Hove, United Kingdom: Psychology Press. 1999. p. 27–44.

[2] Yairi E, Ambrose N. Epidemiology of stuttering: 21st century advances. J Fluency Disord. 2013;38(2):66–87. doi: 10.1016/j.jfludis.2012.11.002 23773662 PMC3687212

[3] Connery A, McCurtin A, Robinson K. The lived experience of stuttering: a synthesis of qualitative studies with implications for rehabilitation. Disabil Rehabil. 2020;42(16):2232–42. doi: 10.1080/09638288.2018.1555623 30696288

[4] Ambrose NG, Yairi E. Normative disfluency data for early childhood stuttering. J Speech Lang Hear Res. 1999;42(4):895–909. doi: 10.1044/jslhr.4204.895 10450909

[5] Froeschels E. Pathology and therapy of stuttering. Nerv Child. 1943;2:148–61.

[6] Staróbole Juste F, Furquim de Andrade CR. Speech disfluency types of fluent and stuttering individuals: age effects. IALP Student Award. Folia Phoniatr Logop. 2011;63(2):57–64. doi: 10.1159/000319913 20926887

[7] Schwartz HD, Conture EG. Subgrouping young stutterers: preliminary behavioral observations. J Speech Hear Res. 1988;31(1):62–71. doi: 10.1044/jshr.3101.62 3352256

[8] Tichenor SE, Yaruss JS. Group experiences and individual differences in stuttering. J Speech Lang Hear Res. 2019;62(12):4335–50. doi: 10.1044/2019_JSLHR-19-00138 31830852

[9] Chang S-E, Guenther FH. Involvement of the cortico-basal ganglia-thalamocortical loop in developmental stuttering. Front Psychol. 2020;10:3088. doi: 10.3389/fpsyg.2019.03088 32047456 PMC6997432

[10] SheikhBahaei S, Millwater M, Maguire GA. Stuttering as a spectrum disorder: a hypothesis. Curr Res Neurobiol. 2023;5:100116. 38020803 10.1016/j.crneur.2023.100116 PMC10663130

[11] Stager SV. Heterogeneity in stuttering. J Fluen Disord. 1990;15(1):9–19.

[12] Yaruss JS. Documenting individual treatment outcomes in stuttering therapy. Contemp Issues Commun Sci Disord. 2004;31:49–57.

[13] Alm PA. Stuttering and the basal ganglia circuits: a critical review of possible relations. J Commun Disord. 2004;37(4):325–69. doi: 10.1016/j.jcomdis.2004.03.001 15159193

[14] Andrews C, O’Brian S, Harrison E, Onslow M, Packman A, Menzies R. Syllable-timed speech treatment for school-age children who stutter: a phase I trial. Lang Speech Hear Serv Sch. 2012;43(3):359–69. doi: 10.1044/0161-1461(2012/11-0038 22562864

[15] Baxter S, Johnson M, Blank L, Cantrell A, Brumfitt S, Enderby P, et al. The state of the art in non-pharmacological interventions for developmental stuttering. Part 1: a systematic review of effectiveness. Int J Lang Commun Disord. 2015;50(5):676–718. doi: 10.1111/1460-6984.12171 26123259 PMC4755200

[16] Block S, Onslow M, Packman A, Dacakis G. Connecting stuttering management and measurement: IV. Predictors of outcome for a behavioural treatment for stuttering. Int J Lang Commun Disord. 2006;41(4):395–406. doi: 10.1080/13682820600623853 16815808

[17] Brignell A, Krahe M, Downes M, Kefalianos E, Reilly S, Morgan AT. A systematic review of interventions for adults who stutter. J Fluency Disord. 2020;64:105766. doi: 10.1016/j.jfludis.2020.105766 32438123

[18] Foundas AL, Bollich AM, Feldman J, Corey DM, Hurley M, Lemen LC, et al. Aberrant auditory processing and atypical planum temporale in developmental stuttering. Neurology. 2004;63(9):1640–6. doi: 10.1212/01.wnl.0000142993.33158.2a 15534249

[19] O’Brian S, Iverach L, Jones M, Onslow M, Packman A, Menzies R. Effectiveness of the Lidcombe Program for early stuttering in Australian community clinics. Int J Speech Lang Pathol. 2013;15(6):593–603. doi: 10.3109/17549507.2013.783112 23691980

[20] Belyk M, Kraft SJ, Brown S. Stuttering as a trait or state - an ALE meta-analysis of neuroimaging studies. Eur J Neurosci. 2015;41(2):275–84. doi: 10.1111/ejn.12765 25350867 PMC13140564

[21] Etchell AC, Civier O, Ballard KJ, Sowman PF. A systematic literature review of neuroimaging research on developmental stuttering between 1995 and 2016. J Fluen Disord. 2018;55:6–45. 10.1016/j.jfludis.2017.03.007 28778745

[22] Neef NE, Anwander A, Friederici AD. The neurobiological grounding of persistent stuttering: from structure to function. Curr Neurol Neurosci Rep. 2015;15(9):63. doi: 10.1007/s11910-015-0579-4 26228377

[23] Brown S, Ingham RJ, Ingham JC, Laird AR, Fox PT. Stuttered and fluent speech production: an ALE meta-analysis of functional neuroimaging studies. Hum Brain Mapp. 2005;25(1):105–17. doi: 10.1002/hbm.20140 15846815 PMC6871755

[24] De Nil LF, Kroll RM, Lafaille SJ, Houle S. A positron emission tomography study of short- and long-term treatment effects on functional brain activation in adults who stutter. J Fluen Disord. 2003;28(4):357–80. 10.1016/j.jfludis.2003.07.002 14643070

[25] De Nil LF, Kroll RM. Understanding the neural basis of treatment using positron emission tomography. In: Bosshardt HG, Yaruss S, Peters HFM, editors. Fluency disorders: theory, research, treatment and self-help. Nijmegen, The Netherlands: Nijmegen University Press. 2001. p. 43–6.

[26] Etchell AC, Johnson BW, Sowman PF. Behavioral and multimodal neuroimaging evidence for a deficit in brain timing networks in stuttering: a hypothesis and theory. Front Hum Neurosci. 2014;8:467. doi: 10.3389/fnhum.2014.00467 25009487 PMC4070061

[27] Frankford SA, Heller Murray ES, Masapollo M, Cai S, Tourville JA, Nieto-Castañón A, et al. The neural circuitry underlying the “Rhythm Effect” in stuttering. J Speech Lang Hear Res. 2021;64(6S):2325–46. doi: 10.1044/2021_JSLHR-20-00328 33887150 PMC8740675

[28] Kell CA, Neumann K, Behrens M, von Gudenberg AW, Giraud A-L. Speaking-related changes in cortical functional connectivity associated with assisted and spontaneous recovery from developmental stuttering. J Fluency Disord. 2018;55:135–44. doi: 10.1016/j.jfludis.2017.02.001 28216127

[29] Lu C, Chen C, Peng D, You W, Zhang X, Ding G, et al. Neural anomaly and reorganization in speakers who stutter: a short-term intervention study. Neurology. 2012;79(7):625–32. doi: 10.1212/WNL.0b013e31826356d2 22875083

[30] Sitek KR, Cai S, Beal DS, Perkell JS, Guenther FH, Ghosh SS. Decreased cerebellar-orbitofrontal connectivity correlates with stuttering severity: Whole-brain functional and structural connectivity associations with persistent developmental stuttering. Front Hum Neurosci. 2016;10(190):1–11. 27199712 10.3389/fnhum.2016.00190 PMC4855981

[31] Toyomura A, Fujii T, Kuriki S. Effect of an 8-week practice of externally triggered speech on basal ganglia activity of stuttering and fluent speakers. NeuroImage. 2015;109:458–68. 25595501 10.1016/j.neuroimage.2015.01.024

[32] Tourville JA, Guenther FH. The DIVA model: a neural theory of speech acquisition and production. Lang Cogn Process. 2011;26(7):952–81. doi: 10.1080/01690960903498424 23667281 PMC3650855

[33] Bohland JW, Guenther FH. An fMRI investigation of syllable sequence production. Neuroimage. 2006;32(2):821–41. doi: 10.1016/j.neuroimage.2006.04.173 16730195

[34] Max L, Guenther FH, Gracco VL, Ghosh SS, Wallace ME. Unstable or insufficiently activated internal models and feedback-biased motor control as sources of dysfluency: a theoretical model of stuttering. CICSD. 2004;31(Spring):105–22. doi: 10.1044/cicsd_31_s_105

[35] van Lieshout PH, Hulstijn W, Peters HF. Speech production in people who stutter: testing the motor plan assembly hypothesis. J Speech Hear Res. 1996;39(1):76–92. doi: 10.1044/jshr.3901.76 8820700

[36] Max L, Daliri A. Limited pre-speech auditory modulation in individuals who stutter: data and hypotheses. J Speech Lang Hear Res. 2019;62(8S):3071–84. doi: 10.1044/2019_JSLHR-S-CSMC7-18-0358 31465711 PMC6813031

[37] Cai S, Beal DS, Ghosh SS, Guenther FH, Perkell JS. Impaired timing adjustments in response to time-varying auditory perturbation during connected speech production in persons who stutter. Brain Lang. 2014;129:24–9. doi: 10.1016/j.bandl.2014.01.002 24486601 PMC3947674

[38] Cai S, Beal DS, Ghosh SS, Tiede MK, Guenther FH, Perkell JS. Weak responses to auditory feedback perturbation during articulation in persons who stutter: evidence for abnormal auditory-motor transformation. PLoS One. 2012;7(7):e41830. doi: 10.1371/journal.pone.0041830 22911857 PMC3402433

[39] Daliri A, Wieland EA, Cai S, Guenther FH, Chang S-E. Auditory-motor adaptation is reduced in adults who stutter but not in children who stutter. Dev Sci. 2018;21(2):10.1111/desc.12521. doi: 10.1111/desc.12521 28256029 PMC5581739

[40] Daliri A, Max L. Stuttering adults’ lack of pre-speech auditory modulation normalizes when speaking with delayed auditory feedback. Cortex. 2018;99:55–68. doi: 10.1016/j.cortex.2017.10.019 29169049 PMC5801108

[41] Foundas AL, Corey DM, Angeles V, Bollich AM, Crabtree–Hartman E, Heilman KM. Atypical cerebral laterality in adults with persistent developmental stuttering. Neurology. 2003;61(10):1378–85. 14638959 10.1212/01.wnl.0000094320.44334.86

[42] Frankford SA, Cai S, Nieto-Castañón A, Guenther FH. Auditory feedback control in adults who stutter during metronome-paced speech II. Formant Perturbation. J Fluency Disord. 2022;74:105928. doi: 10.1016/j.jfludis.2022.105928 36063640 PMC9930613

[43] Kim KS, Daliri A, Flanagan JR, Max L. Dissociated development of speech and limb sensorimotor learning in stuttering: speech auditory-motor learning is impaired in both children and adults who stutter. Neuroscience. 2020;451:1–21. doi: 10.1016/j.neuroscience.2020.10.014 33091464 PMC7704609

[44] Sares AG, Deroche MLD, Shiller DM, Gracco VL. Timing variability of sensorimotor integration during vocalization in individuals who stutter. Sci Rep. 2018;8(1):16340. doi: 10.1038/s41598-018-34517-1 30397215 PMC6218511

[45] Sengupta R, Shah S, Gore K, Loucks T, Nasir SM. Anomaly in neural phase coherence accompanies reduced sensorimotor integration in adults who stutter. Neuropsychologia. 2016;93(Pt A):242–50. doi: 10.1016/j.neuropsychologia.2016.11.004 27833009

[46] De Nil LF, Kroll RM, Houle S. Functional neuroimaging of cerebellar activation during single word reading and verb generation in stuttering and nonstuttering adults. Neurosci Lett. 2001;302(2–3):77–80. doi: 10.1016/s0304-3940(01)01671-8 11290391

[47] Fukawa T, Yoshioka H, Ozawa E, Yoshida S. Difference of susceptibility to delayed auditory feedback between stutterers and nonstutterers. J Speech Hear Res. 1988;31(3):475–9. doi: 10.1044/jshr.3103.475 3172765

[48] Iimura D, Asakura N, Sasaoka T, Inui T. Abnormal sensorimotor integration in adults who stutter: a behavioral study by adaptation of delayed auditory feedback. Front Psychol. 2019;10:2440. doi: 10.3389/fpsyg.2019.02440 31736833 PMC6834693

[49] Cai S, Tourville JA, Beal DS, Perkell JS, Guenther FH, Ghosh SS. Diffusion imaging of cerebral white matter in persons who stutter: evidence for network-level anomalies. Front Hum Neurosci. 2014;8:54. doi: 10.3389/fnhum.2014.00054 24611042 PMC3920071

[50] Korzeczek A, Primaßin A, Wolff von Gudenberg A, Dechent P, Paulus W, Sommer M, et al. Fluency shaping increases integration of the command-to-execution and the auditory-to-motor pathways in persistent developmental stuttering. Neuroimage. 2021;245:118736. doi: 10.1016/j.neuroimage.2021.118736 34798230

[51] Sares AG, Deroche MLD, Ohashi H, Shiller DM, Gracco VL. Neural correlates of vocal pitch compensation in individuals who stutter. Front Hum Neurosci. 2020;14:18. doi: 10.3389/fnhum.2020.00018 32161525 PMC7053555

[52] Foundas AL, Mock JR, Cindass R Jr, Corey DM. Atypical caudate anatomy in children who stutter. Percept Mot Skills. 2013;116(2):528–43. doi: 10.2466/15.10.PMS.116.2.528-543 24032328

[53] Guenther FH. Neural control of speech. Cambridge, Massachusetts: The MIT Press. 2016.

[54] Jiang J, Lu C, Peng D, Zhu C, Howell P. Classification of types of stuttering symptoms based on brain activity. PLoS One. 2012;7(6):e39747. doi: 10.1371/journal.pone.0039747 22761887 PMC3382568

[55] Altrows IF, Bryden MP. Temporal factors in the effects of masking noise on fluency of stutterers. J Commun Disord. 1977;10(4):315–29. 925194 10.1016/0021-9924(77)90029-6

[56] Conture E, Brayton E. The influence of noise on stutterers’ different disfluency types. J Speech Lang Hear Res. 1975;18:381.

[57] Hutchinson JM, Norris GM. The differential effect of three auditory stimuli on the frequency of stuttering behaviors. J Fluen Disord. 1977;2(4):283–93.

[58] Silverman FH, Goodban MT. The effect of auditory masking on the fluency of normal speakers. J Speech Hear Res. 1972;15(3):543–6. doi: 10.1044/jshr.1503.543 5080045

[59] Civier O, Tasko SM, Guenther FH. Overreliance on auditory feedback may lead to sound/syllable repetitions: simulations of stuttering and fluency-inducing conditions with a neural model of speech production. J Fluency Disord. 2010;35(3):246–79. doi: 10.1016/j.jfludis.2010.05.002 20831971 PMC2939043

[60] Harrington J. Coarticulation and stuttering: an acoustic and electropalatographic study. In: Peters HFM, Hulstijn W, editors. Speech motor dynamics in stuttering. New York: Springer-Verlag. 1987. p. 381–92.

[61] Howell P, Vause L. Acoustic analysis and perception of vowels in stuttered speech. J Acoust Soc Am. 1986;79(5):1571–9. 3711457 10.1121/1.393684

[62] Stromsta C. Elements of stuttering. Oshtemo, MI: Atsmorts. 1986.

[63] Yaruss JS, Conture EG. F2 transitions during sound/syllable repetitions of children who stutter and predictions of stuttering chronicity. J Speech Hear Res. 1993;36(5):883–96. doi: 10.1044/jshr.3605.883 8246477

[64] Zimmermann G. Articulatory behaviors associated with stuttering: a cinefluorographic analysis. J Speech Hear Res. 1980;23(1):108–21. doi: 10.1044/jshr.2301.108 7442171

[65] Adams MR, Hutchinson J. The effects of three levels of auditory masking on selected vocal characteristics and the frequency of disfluency of adult stutterers. J Speech Hear Res. 1974;17(4):682–8. doi: 10.1044/jshr.1704.682 4444288

[66] Maraist JA, Hutton C. Effects of auditory masking upon the speech of stutterers. J Speech Hear Disord. 1957;22(3):385–9. doi: 10.1044/jshd.2203.385 13463921

[67] Civier O, Bullock D, Max L, Guenther FH. Simulating neural impairments to syllable-level command generation in stuttering. In: 6th World Congress on Fluency Disorders. 2009.

[68] Civier O, Bullock D, Max L, Guenther FH. Computational modeling of stuttering caused by impairments in a basal ganglia thalamo-cortical circuit involved in syllable selection and initiation. Brain Lang. 2013;126(3):263–78. doi: 10.1016/j.bandl.2013.05.016 23872286 PMC3775364

[69] Mennes M, Vega Potler N, Kelly C, Di Martino A, Castellanos FX, Milham MP. Resting state functional connectivity correlates of inhibitory control in children with attention-deficit/hyperactivity disorder. Front Psychiatry. 2012;2:83. doi: 10.3389/fpsyt.2011.00083 22470352 PMC3261661

[70] Finn ES, Shen X, Scheinost D, Rosenberg MD, Huang J, Chun MM, et al. Functional connectome fingerprinting: identifying individuals using patterns of brain connectivity. Nat Neurosci. 2015;18(11):1664–71. doi: 10.1038/nn.4135 26457551 PMC5008686

[71] Viviano JD, Buchanan RW, Calarco N, Gold JM, Foussias G, Bhagwat N, et al. Resting-state connectivity biomarkers of cognitive performance and social function in individuals with schizophrenia spectrum disorder and healthy control subjects. Biol Psychiatry. 2018;84(9):665–74. doi: 10.1016/j.biopsych.2018.03.013 29779671 PMC6177285

[72] Bernard JA, Seidler RD, Hassevoort KM, Benson BL, Welsh RC, Wiggins JL, et al. Resting state cortico-cerebellar functional connectivity networks: a comparison of anatomical and self-organizing map approaches. Front Neuroanat. 2012;6:31. doi: 10.3389/fnana.2012.00031 22907994 PMC3415673

[73] Mariën P, Ackermann H, Adamaszek M, Barwood CHS, Beaton A, Desmond J, et al. Consensus paper: language and the cerebellum: an ongoing enigma. Cerebellum. 2014;13(3):386–410. doi: 10.1007/s12311-013-0540-5 24318484 PMC4090012

[74] Bloodstein O, Bernstein Ratner N. A handbook on stuttering. 6th ed. New York, NY: Thomson-Delmar. 2008.

[75] Oldfield RC. The assessment and analysis of handedness: the Edinburgh inventory. Neuropsychologia. 1971;9(1):97–113. doi: 10.1016/0028-3932(71)90067-4 5146491

[76] Riley GD. Stuttering severity instrument for children and adults (SSI-4). Austin, TX: Pro-Ed Publishers, Inc.; 2009.

[77] Van Riper C. Speech correction. 4th ed. Englewood Cliffs, NJ: Prentice Hall. 1963.

[78] Guitar B. Stuttering: an integrated approach to its nature and treatment. 5th ed. Philadelphia, PA: Lippincott Williams & Wilkins. 2019.

[79] Whitfield-Gabrieli S, Nieto-Castanon A. Conn: a functional connectivity toolbox for correlated and anticorrelated brain networks. Brain Connect. 2012;2(3):125–41. doi: 10.1089/brain.2012.0073 22642651

[80] Morfini F, Whitfield-Gabrieli S, Nieto-Castañón A. Functional connectivity MRI quality control procedures in CONN. Front Neurosci. 2023;17:1092125. 37034165 10.3389/fnins.2023.1092125 PMC10076563

[81] Nieto-Castanon A. Handbook of functional connectivity magnetic resonance imaging methods in CONN. Hilbert Press. 2020.

[82] Coalson TS, Van Essen DC, Glasser MF. The impact of traditional neuroimaging methods on the spatial localization of cortical areas. Proc Natl Acad Sci. 2018;115(27). 10.1073/pnas.1801582115 PMC6142239 29925602

[83] Fischl B, Sereno MI, Dale AM. Cortical surface-based analysis: II. Inflation, flattening, and a surface-based coordinate system. NeuroImage. 1999;9:195–207. 9931269 10.1006/nimg.1998.0396

[84] Van Essen DC. A Population-Average, Landmark- and Surface-based (PALS) atlas of human cerebral cortex. Neuroimage. 2005;28(3):635–62. doi: 10.1016/j.neuroimage.2005.06.058 16172003

[85] Glasser MF, Coalson TS, Robinson EC, Hacker CD, Harwell J, Yacoub E, et al. A multi-modal parcellation of human cerebral cortex. Nature. 2016;536(7615):171–8. doi: 10.1038/nature18933 27437579 PMC4990127

[86] Behzadi Y, Restom K, Liau J, Liu TT. A component based noise correction method (CompCor) for BOLD and perfusion based fMRI. Neuroimage. 2007;37(1):90–101. doi: 10.1016/j.neuroimage.2007.04.042 17560126 PMC2214855

[87] Andersson JL, Hutton C, Ashburner J, Turner R, Friston K. Modeling geometric deformations in EPI time series. Neuroimage. 2001;13(5):903–19. doi: 10.1006/nimg.2001.0746 11304086

[88] Nieto-Castanon A. CONN functional connectivity toolbox (RRIDSCR_009). Hilbert Press. 2020.

[89] Ségonne F, Dale AM, Busa E, Glessner M, Salat D, Hahn HK, et al. A hybrid approach to the skull stripping problem in MRI. Neuroimage. 2004;22(3):1060–75. doi: 10.1016/j.neuroimage.2004.03.032 15219578

[90] Dale AM, Fischl B, Sereno MI. Cortical surface-based analysis. I. Segmentation and surface reconstruction. Neuroimage. 1999;9(2):179–94. doi: 10.1006/nimg.1998.0395 9931268

[91] Ségonne F, Pacheco J, Fischl B. Geometrically accurate topology-correction of cortical surfaces using nonseparating loops. IEEE Trans Med Imaging. 2007;26(4):518–29. doi: 10.1109/TMI.2006.887364 17427739

[92] Hagler DJ Jr, Saygin AP, Sereno MI. Smoothing and cluster thresholding for cortical surface-based group analysis of fMRI data. Neuroimage. 2006;33(4):1093–103. doi: 10.1016/j.neuroimage.2006.07.036 17011792 PMC1785301

[93] Ashburner J, Friston KJ. Unified segmentation. Neuroimage. 2005;26(3):839–51. doi: 10.1016/j.neuroimage.2005.02.018 15955494

[94] Krauth A, Blanc R, Poveda A, Jeanmonod D, Morel A, Székely G. A mean three-dimensional atlas of the human thalamus: generation from multiple histological data. Neuroimage. 2010;49(3):2053–62. doi: 10.1016/j.neuroimage.2009.10.042 19853042

[95] Keuken MC, Bazin P-L, Crown L, Hootsmans J, Laufer A, Müller-Axt C, et al. Quantifying inter-individual anatomical variability in the subcortex using 7 T structural MRI. Neuroimage. 2014;94:40–6. doi: 10.1016/j.neuroimage.2014.03.032 24650599

[96] Fisher NI, Hall P. Bootstrap algorithms for small samples. J Stat Plan Inference. 1991;27(2):157–69.

[97] Tibshirani R. Regression shrinkage and selection via the lasso. J R Stat Soc Ser B Stat Methodol. 1996;58(1):267–88.

[98] Meinshausen N, Bühlmann P. Stability selection. J R Stat Soc Ser B Stat Methodol. 2010;72(4):417–73.

[99] Chang S-E, Garnett EO, Etchell A, Chow HM. Functional and neuroanatomical bases of developmental stuttering: current insights. Neuroscientist. 2019;25(6):566–82. doi: 10.1177/1073858418803594 30264661 PMC6486457

[100] Chow HM, Garnett EO, Ratner NB, Chang S-E. Brain activity during the preparation and production of spontaneous speech in children with persistent stuttering. Neuroimage Clin. 2023;38:103413. doi: 10.1016/j.nicl.2023.103413 37099876 PMC10149502

[101] Diedrichsen J, Verstynen T, Lehman SL, Ivry RB. Cerebellar involvement in anticipating the consequences of self-produced actions during bimanual movements. J Neurophysiol. 2005;93(2):801–12. doi: 10.1152/jn.00662.2004 15356182

[102] Grafton ST, Schmitt P, Van Horn J, Diedrichsen J. Neural substrates of visuomotor learning based on improved feedback control and prediction. Neuroimage. 2008;39(3):1383–95. doi: 10.1016/j.neuroimage.2007.09.062 18032069 PMC2268716

[103] Penhune VB, Doyon J. Cerebellum and M1 interaction during early learning of timed motor sequences. Neuroimage. 2005;26(3):801–12. doi: 10.1016/j.neuroimage.2005.02.041 15955490

[104] Runnqvist E, Chanoine V, Strijkers K, Pattamadilok C, Bonnard M, Nazarian B, et al. Cerebellar and cortical correlates of internal and external speech error monitoring. Cereb Cortex Commun. 2021;2(2):tgab038. doi: 10.1093/texcom/tgab038 34296182 PMC8237718

[105] Manto M, Bower JM, Conforto AB, Delgado-García JM, da Guarda SNF, Gerwig M, et al. Consensus paper: roles of the cerebellum in motor control--the diversity of ideas on cerebellar involvement in movement. Cerebellum. 2012;11(2):457–87. doi: 10.1007/s12311-011-0331-9 22161499 PMC4347949

[106] O’Reilly JX, Beckmann CF, Tomassini V, Ramnani N, Johansen-Berg H. Distinct and overlapping functional zones in the cerebellum defined by resting state functional connectivity. Cereb Cortex. 2010;20(4):953–65. doi: 10.1093/cercor/bhp157 19684249 PMC2837094

[107] Dick AS, Garic D, Graziano P, Tremblay P. The frontal aslant tract (FAT) and its role in speech, language and executive function. Cortex. 2019;111:148–63. 30481666 10.1016/j.cortex.2018.10.015 PMC6461388

[108] Akkal D, Dum RP, Strick PL. Supplementary motor area and presupplementary motor area: targets of basal ganglia and cerebellar output. J Neurosci. 2007;27(40):10659–73. doi: 10.1523/JNEUROSCI.3134-07.2007 17913900 PMC6672811

[109] Metzger FL, Auer T, Helms G, Paulus W, Frahm J, Sommer M, et al. Shifted dynamic interactions between subcortical nuclei and inferior frontal gyri during response preparation in persistent developmental stuttering. Brain Struct Funct. 2018;223(1):165–82. doi: 10.1007/s00429-017-1476-1 28741037 PMC5772149

[110] Rowe HP, Tourville JA, Nieto-Castanon A, Garnett EO, Chow HM, Chang S-E, et al. Evidence for planning and motor subtypes of stuttering based on resting state functional connectivity. Brain Lang. 2024;253:105417. doi: 10.1016/j.bandl.2024.105417 38703523 PMC11147703

[111] Neef NE, Anwander A, Bütfering C, Schmidt-Samoa C, Friederici AD, Paulus W, et al. Structural connectivity of right frontal hyperactive areas scales with stuttering severity. Brain. 2018;141(1):191–204. doi: 10.1093/brain/awx316 29228195 PMC5837552

[112] Mailend ML, Maas E, Beeson PM, Story BH, Forster KI. Speech motor planning in the context of phonetically similar words: evidence from apraxia of speech and aphasia. Neuropsychologia. 2019;127:171–84. 30817912 10.1016/j.neuropsychologia.2019.02.018 PMC6459184

[113] Miller HE, Guenther FH. Modelling speech motor programming and apraxia of speech in the DIVA/GODIVA neurocomputational framework. Aphasiology. 2021;35(4):424–41. doi: 10.1080/02687038.2020.1765307 34108793 PMC8183977

[114] Postma A, Kolk H. The covert repair hypothesis: prearticulatory repair processes in normal and stuttered disfluencies. J Speech Hear Res. 1993;36(3):472–87. 8331905

[115] Conture EG, Walden TA. Dual diathesis-stressor model of stuttering. In: Filatova YO, editor. Theoretical issues of fluency disorders. Moscow: National Book Centre. 2012. p. 94–127.

[116] Einarsdóttir J, Ingham RJ. Have disfluency-type measures contributed to the understanding and treatment of developmental stuttering? Am J Speech Lang Pathol. 2005;14(4):260–73. doi: 10.1044/1058-0360(2005/026 16396610

[117] Bothe AK. Identification of children’s stuttered and nonstuttered speech by highly experienced judges: binary judgments and comparisons with disfluency-types definitions. J Speech Lang Hear Res. 2008;51(4):867–78. 18658057 10.1044/1092-4388(2008/063)

[118] Jackson ES, Gracco V, Zebrowski PM. Eliciting stuttering in laboratory contexts. J Speech Lang Hear Res. 2019;63(1):143–50. doi: 10.1044/2019_JSLHR-S-19-0173 31835000 PMC7213478

[119] Chesters J, Mottonen R, Watkins KE. Transcranial direct current stimulation over left inferior frontal cortex improves speech fluency in adults who stutter. Brain. 2018;141:1161–71. 29394325 10.1093/brain/awy011 PMC6019054

[120] Garnett EO, Chow HM, Choo AL, Chang S-E. Stuttering severity modulates effects of non-invasive brain stimulation in adults who stutter. Front Hum Neurosci. 2019;13:411. doi: 10.3389/fnhum.2019.00411 31824276 PMC6881273

[121] Maguire GA, Riley GD, Franklin DL, Gottschalk LA. Risperidone for the treatment of stuttering. J Clin Psychopharmacol. 2000;20(4):479–82. doi: 10.1097/00004714-200008000-00013 10917410

[122] Wu JC, Maguire G, Riley G, Lee A, Keator D, Tang C, et al. Increased dopamine activity associated with stuttering. Neuroreport. 1997;8(3):767–70. doi: 10.1097/00001756-199702100-00037 9106763

