# Supplementary material for: Differential involvement of feedback and feedforward control networks across disfluency types in adults who stutter: Evidence from resting state functional connectivity
Source: PLoS One. 2025 Sep 26;20(9):e0333205. doi: 10.1371/journal.pone.0333205 (PMC12468764; doi:10.1371/journal.pone.0333205)
Supplement: Table S1 — (DOCX) [file pone.0333205.s001.docx]

**Table S1.** Results of Levene’s tests assessing potential heteroscedasticity due to inter-site variance.

| **Connection** | ***Df1*** | ***Df2*** | ***F*** | ***p*** | ***p* (FDR-adjusted)** |
| --- | --- | --- | --- | --- | --- |
| Right aCbm - Left MG | 1 | 18 | 0.1416502295 | 0.71104552 | 0.9476729 |
| Right pCbm - Left MG | 1 | 18 | 4.9970673967 | 0.03829992 | 0.8965542 |
| Right aCbm - Left VPM | 1 | 18 | 0.0003422613 | 0.98544326 | 0.9893365 |
| Right pCbm - Left VPM | 1 | 18 | 1.5712849315 | 0.22605823 | 0.8965542 |
| Left VA - Left SMA | 1 | 18 | 3.6714548643 | 0.07137209 | 0.8965542 |
| Left VL - Left SMA | 1 | 18 | 5.4397920776 | 0.03149295 | 0.8965542 |
| Left VA - Left vPMC | 1 | 18 | 0.2243511887 | 0.64143848 | 0.9476729 |
| Left VL - Left vPMC | 1 | 18 | 0.4972305033 | 0.48974465 | 0.8965542 |
| Left SMA - Left vPMC | 1 | 18 | 1.1376606711 | 0.30024511 | 0.8965542 |
| Left VA - Left Pallidum | 1 | 18 | 0.4397681665 | 0.51563788 | 0.8965542 |
| Left VL - Left Pallidum | 1 | 18 | 0.5311297433 | 0.4755084 | 0.8965542 |
| Left VA - Left vMC | 1 | 18 | 0.2243511887 | 0.64143848 | 0.9476729 |
| Left VL - Left vMC | 1 | 18 | 0.4972305033 | 0.48974465 | 0.8965542 |
| Right aCbm - Left vPMC | 1 | 18 | 0.4420432289 | 0.51456671 | 0.8965542 |
| Right pCbm - Left vPMC | 1 | 18 | 1.5055357749 | 0.235629 | 0.8965542 |
| Right aCbm - Left VL | 1 | 18 | 0.1930346074 | 0.66563196 | 0.9476729 |
| Right pCbm - Left VL | 1 | 18 | 0.723294465 | 0.40623669 | 0.8965542 |
| Left VL - Right LobuleVI | 1 | 18 | 3.2765740806 | 0.08700321 | 0.8965542 |
| Left vSC - Left vPMC | 1 | 18 | 2.6237303795 | 0.12266368 | 0.8965542 |
| Left vSC - Right vPMC | 1 | 18 | 0.7846813466 | 0.3873946 | 0.8965542 |
| Right aCbm - Right vPMC | 1 | 18 | 0.1737436626 | 0.68173461 | 0.9476729 |
| Right pCbm - Right vPMC | 1 | 18 | 2.3505392444 | 0.1426279 | 0.8965542 |
| Left vMC - Right vPMC | 1 | 18 | 0.4723966821 | 0.50064734 | 0.8965542 |
| Left H - Left vPMC | 1 | 18 | 0.1370230053 | 0.71557837 | 0.9476729 |
| Left PT - Left vPMC | 1 | 18 | 0.0170767224 | 0.89747949 | 0.9893365 |
| Left pSTG - Left vPMC | 1 | 18 | 1.3478254353 | 0.2608243 | 0.8965542 |
| Left MG - Left H | 1 | 18 | 0.0052536316 | 0.94301771 | 0.9893365 |
| Left MG - Left PT | 1 | 18 | 0.3933478025 | 0.53841973 | 0.8965542 |
| Left MG - Left pSTG | 1 | 18 | 0.3882730263 | 0.54102408 | 0.8965542 |
| Left PT - Right vPMC | 1 | 18 | 0.0297873425 | 0.86489943 | 0.9893365 |
| Left pSTG - Right vPMC | 1 | 18 | 1.1102700004 | 0.30596284 | 0.8965542 |
| Left H - Right vPMC | 1 | 18 | 0.1568320748 | 0.69674501 | 0.9476729 |
| Left Putamen - Left Pallidum | 1 | 18 | 0.0093758701 | 0.92393212 | 0.9893365 |
| Left VPM - Left vSC | 1 | 18 | 0.0838509404 | 0.77545406 | 0.9569433 |
| Left vMC - Left vPMC | 1 | 18 | 0.8700888373 | 0.36327503 | 0.8965542 |
| Left Putamen - Left vPMC | 1 | 18 | 1.4578375146 | 0.24290144 | 0.8965542 |
| Left Putamen - Left vMC | 1 | 18 | 0.1233402065 | 0.72951682 | 0.9476729 |
| Left preSMA - Left pIFS | 1 | 18 | 0.6931609292 | 0.41600152 | 0.8965542 |
| Left Caudate - Left Pallidum | 1 | 18 | 0.7157831799 | 0.40863749 | 0.8965542 |
| Left Caudate - Left pIFS | 1 | 18 | 0.9738460287 | 0.33680324 | 0.8965542 |
| Left Caudate - Left preSMA | 1 | 18 | 0.8430929393 | 0.37065417 | 0.8965542 |
| Left VA - Left preSMA | 1 | 18 | 0.4808770187 | 0.49687703 | 0.8965542 |
| Left VA - Left pIFS | 1 | 18 | 0.1033037061 | 0.75160268 | 0.9476729 |
| Left Putamen - Left SMA | 1 | 18 | 0.0029744941 | 0.95710658 | 0.9893365 |
| Left SMA - Left preSMA | 1 | 18 | 1.3377797595 | 0.26254621 | 0.8965542 |
| Left pIFS - Left vPMC | 1 | 18 | 2.1768692589 | 0.15737755 | 0.8965542 |
| Right PT - Right vPMC | 1 | 18 | 1.2283976171 | 0.2823091 | 0.8965542 |
| Right PT - Left vPMC | 1 | 18 | 1.510128525 | 0.2349437 | 0.8965542 |
| Right PT - Right MG | 1 | 18 | 0.0411705765 | 0.84148498 | 0.9893365 |
| Right PT - Left MG | 1 | 18 | 0.3064560916 | 0.58667446 | 0.9451977 |
| Right pSTG - Right vPMC | 1 | 18 | 0.4062161513 | 0.53192023 | 0.8965542 |
| Right pSTG - Left vPMC | 1 | 18 | 0.0192465424 | 0.89120233 | 0.9893365 |
| Right pSTG - Right MG | 1 | 18 | 1.585692188 | 0.22402807 | 0.8965542 |
| Right pSTG - Left MG | 1 | 18 | 0.6308942615 | 0.43737489 | 0.8965542 |
| Right H - Right vPMC | 1 | 18 | 0.0081336925 | 0.92913461 | 0.9893365 |
| Right H - Left vPMC | 1 | 18 | 0.0595332748 | 0.80999454 | 0.9787434 |
| Right H - Right MG | 1 | 18 | 0.0001836558 | 0.98933651 | 0.9893365 |
| Right H - Left MG | 1 | 18 | 0.1045965575 | 0.75010899 | 0.9476729 |
| a/pCb = anterior/posterior cerebellum; GP = globus pallidus; H = Heschl’s gyrus; MG = medial geniculate thalamic nucleus; pAC = posterior auditory cortex; pIFS = posterior inferior frontal sulcus; preSMA = presupplementary motor area; pSTG = posterior superior temporal cortex; PT = planum temporale; SMA = supplementary motor area; VA = ventral anterior thalamic nucleus; VL = ventral lateral thalamic nucleus; vMC = ventral motor cortex; vPMC = ventral premotor cortex; VPM = ventral posterior medial thalamic nucleus; vSC = ventral somatosensory cortex. | | | | | |
