# Supplementary material for: Differential involvement of feedback and feedforward control networks across disfluency types in adults who stutter: Evidence from resting state functional connectivity
Source: PLoS One. 2025 Sep 26;20(9):e0333205. doi: 10.1371/journal.pone.0333205 (PMC12468764; doi:10.1371/journal.pone.0333205)
Supplement: Table S2 — (DOCX) [file pone.0333205.s002.docx]

**Table S2.** Coefficient estimates, standard errors, and 95% confidence intervals of connections with statistically significant effects identified by each LASSO regression.

| **Stuttering Type** | **Connection** | **Estimate** | **Standard Error** | **Lower CI** | **Upper CI** |
| --- | --- | --- | --- | --- | --- |
| Repetitions | Left vMC - Right vPMC | 0.192563807 | 0.008005935 | 0.1609532872 | 0.2241743276 |
| Repetitions | Left SMA - Left vPMC | -0.092562993 | 0.0045252879 | -0.1104305755 | -0.0746954108 |
| Repetitions | Left MG - Left pSTG | 0.089460249 | 0.0050891627 | 0.0693662714 | 0.1095542272 |
| Repetitions | Left Caudate - Left pIFS | 0.080850569 | 0.0045612099 | 0.0628411523 | 0.0988599848 |
| Repetitions | Left PT - Right vPMC | -0.051248211 | 0.0032148558 | -0.0639417013 | -0.0385547197 |
| Repetitions | Right pCbm - Left MG | 0.040960773 | 0.0029537732 | 0.0292981369 | 0.0526234091 |
| Repetitions | Left vMC - Left vPMC | -0.037065122 | 0.0030739448 | -0.0492022414 | -0.0249280016 |
| Repetitions | Left VA - Left vPMC | -0.032792446 | 0.0024173542 | -0.0423370923 | -0.0232477987 |
| Repetitions | Right aCbm - Right vPMC | -0.031715753 | 0.0028435907 | -0.0429433464 | -0.0204881601 |
| Repetitions | Left preSMA - Left pIFS | 0.031357309 | 0.0023927522 | 0.0219098002 | 0.0408048177 |
| Repetitions | Left pSTG - Right vPMC | 0.022384417 | 0.0027390328 | 0.0115696594 | 0.0331991756 |
| Repetitions | Right pCbm - Left vPMC | -0.020233494 | 0.0023714853 | -0.0295970325 | -0.0108699551 |
| Repetitions | Left MG - Left H | 0.01924049 | 0.0021784192 | 0.010639251 | 0.0278417295 |
| Repetitions | Left VL - Left vPMC | -0.018354636 | 0.002165469 | -0.0269047428 | -0.0098045286 |
| Repetitions | Left VL - Left Pallidum | -0.018097382 | 0.0021991119 | -0.0267803246 | -0.0094144403 |
| Repetitions | Left H - Right vPMC | -0.01783266 | 0.0020570116 | -0.0259545357 | -0.009710785 |
| Repetitions | Left VA - Left preSMA | -0.015022882 | 0.0022279159 | -0.0238195534 | -0.0062262106 |
| Repetitions | Right PT - Right vPMC | -0.013578186 | 0.0016528834 | -0.0201044071 | -0.0070519641 |
| Repetitions | Left VL - Left SMA | -0.010367722 | 0.0016688981 | -0.016957175 | -0.0037782682 |
| Repetitions | Right H - Right vPMC | -0.007025475 | 0.001116802 | -0.0114350405 | -0.0026159103 |
| Repetitions | Right pSTG - Left vPMC | -0.006640062 | 0.0011242512 | -0.0110790391 | -0.0022010844 |
| Repetitions | Left Caudate - Left preSMA | 0.006243085 | 0.0010482118 | 0.0021043406 | 0.0103818299 |
| Repetitions | Left Putamen - Left Pallidum | -0.004579237 | 0.0009210165 | -0.0082157656 | -0.0009427084 |
| Repetitions | Left Putamen - Left SMA | -0.004570766 | 0.0010143987 | -0.0085760033 | -0.0005655285 |
| Repetitions | Left vSC - Left vPMC | -0.003017199 | 0.0006461716 | -0.0055685333 | -0.0004658639 |
| Repetitions | Left pIFS - Left vPMC | 0.002919836 | 0.0006591252 | 0.0003173551 | 0.0055223164 |
| Repetitions | Right aCbm - Left MG | 0.00243668 | 0.000605508 | 0.0000459006 | 0.0048274591 |
| Prolongations | Left vMC - Right vPMC | 0.03997022 | 0.0027629761 | 0.02906092436 | 0.0508795154 |
| Prolongations | Left MG - Left H | 0.024817195 | 0.0021432067 | 0.01635498773 | 0.0332794016 |
| Prolongations | Left SMA - Left vPMC | -0.014091218 | 0.0015659593 | -0.020274229 | -0.0079082062 |
| Prolongations | Left VL - Left Pallidum | -0.013612005 | 0.0015120877 | -0.01958231032 | -0.0076416994 |
| Prolongations | Left vMC - Left vPMC | -0.013001717 | 0.0013425833 | -0.01830275369 | -0.0077006799 |
| Prolongations | Right PT - Left vPMC | 0.012688282 | 0.0013632116 | 0.00730579662 | 0.0180707673 |
| Prolongations | Right aCbm - Left MG | 0.012056105 | 0.0012475536 | 0.00713028237 | 0.0169819281 |
| Prolongations | Right pCbm - Left VL | -0.010470896 | 0.0012803291 | -0.0155261296 | -0.0054156633 |
| Prolongations | Left PT - Right vPMC | -0.010470739 | 0.0015063459 | -0.01641837346 | -0.0045231043 |
| Prolongations | Right pSTG - Right vPMC | 0.008673938 | 0.0011541071 | 0.00411707832 | 0.0132307982 |
| Prolongations | Left vSC - Right vPMC | 0.006148271 | 0.0009782856 | 0.00228562209 | 0.0100109197 |
| Prolongations | Left Caudate - Left pIFS | 0.005991917 | 0.0008816558 | 0.00251080003 | 0.0094730343 |
| Prolongations | Left VA - Left vPMC | -0.005792947 | 0.0009245115 | -0.00944327532 | -0.0021426192 |
| Prolongations | Right pSTG - Left vPMC | 0.005718675 | 0.0009606654 | 0.00192559682 | 0.0095117524 |
| Prolongations | Left VA - Left SMA | -0.005268838 | 0.0011390064 | -0.00976607473 | -0.0007716012 |
| Prolongations | Left MG - Left pSTG | 0.004647695 | 0.0008628062 | 0.00124100321 | 0.0080543866 |
| Prolongations | Right pCbm - Right vPMC | 0.004476669 | 0.0008237169 | 0.00122431723 | 0.0077290212 |
| Prolongations | Left VA - Left preSMA | -0.004018985 | 0.0007589973 | -0.00701579918 | -0.0010221705 |
| Prolongations | Right aCbm - Left VPM | 0.003724763 | 0.0006472608 | 0.00116912783 | 0.0062803989 |
| Prolongations | Right H - Left vPMC | 0.003294061 | 0.0006542531 | 0.00071081746 | 0.005877305 |
| Prolongations | Right pCbm - Left MG | 0.003146754 | 0.0006147013 | 0.00071967626 | 0.005573832 |
| Prolongations | Left Putamen - Left vPMC | 0.002068416 | 0.0004960681 | 0.00010974753 | 0.004027084 |
| Prolongations | Right aCbm - Left VL | 0.001960564 | 0.0004728661 | 0.00009350612 | 0.0038276217 |
| Prolongations | Right aCbm - Right vPMC | 0.001550169 | 0.0003589791 | 0.00013278138 | 0.0029675576 |
| Prolongations | Right aCbm - Left vPMC | 0.00151256 | 0.0003218263 | 0.00024186532 | 0.0027832545 |
| Blocks | Right aCbm - Left VL | -0.16191528 | 0.015213952 | -0.2219858304 | -0.1018447259 |
| Blocks | Left preSMA - Left pIFS | 0.11846216 | 0.011364514 | 0.0735906703 | 0.1633336421 |
| Blocks | Left MG - Left H | 0.10710829 | 0.012857993 | 0.0563399724 | 0.1578766057 |
| Blocks | Left vMC - Right vPMC | 0.10671361 | 0.012145727 | 0.0587575902 | 0.1546696253 |
| Blocks | Right pCbm - Right vPMC | 0.07818873 | 0.010110416 | 0.0382689031 | 0.1181085529 |
| Blocks | Right pCbm - Left VL | -0.06859161 | 0.008442535 | -0.101925994 | -0.0352572249 |
| Blocks | Left SMA - Left vPMC | -0.06344495 | 0.008533894 | -0.0971400574 | -0.0297498478 |
| Blocks | Left MG - Left pSTG | 0.04979685 | 0.007577673 | 0.0198772777 | 0.0797164315 |
| Blocks | Right pCbm - Left vPMC | 0.04505481 | 0.007377685 | 0.0159248688 | 0.0741847583 |
| Blocks | Left VA - Left SMA | 0.04401768 | 0.007042713 | 0.0162103375 | 0.0718250323 |
| Blocks | Left pIFS -Left vPMC | -0.03633615 | 0.006216047 | -0.0608794967 | -0.0117927964 |
| Blocks | Right aCbm - Left vPMC | -0.03631201 | 0.004867527 | -0.0555308874 | -0.0170931408 |
| Blocks | Right aCbm - Left MG | -0.0353349 | 0.005694506 | -0.057819006 | -0.0128507956 |
| Blocks | Left vSC - Left vPMC | -0.03515287 | 0.005365885 | -0.056339456 | -0.013966285 |
| Blocks | Right H - Left MG | 0.02851327 | 0.004681486 | 0.0100289561 | 0.04699758 |
| Blocks | Left Putamen - Left vPMC | -0.02704923 | 0.004566643 | -0.0450800953 | -0.0090183615 |
| Blocks | Left VA - Left preSMA | 0.02134243 | 0.004893123 | 0.0020224937 | 0.0406623671 |
| Blocks | Left pSTG - Right vPMC | 0.02028003 | 0.004410109 | 0.0028672239 | 0.037692845 |
| Blocks | Left VA - Left vPMC | -0.0162296 | 0.003381041 | -0.0295792517 | -0.0028799414 |
| Blocks | Right pSTG - Right MG | 0.01596482 | 0.003298599 | 0.0029406745 | 0.0289889592 |
| Blocks | Left VL - Right LobuleVI | -0.01310435 | 0.003144983 | -0.0255219534 | -0.0006867386 |
| Blocks | Right PT - Left vPMC | 0.00967344 | 0.002323984 | 0.0004974555 | 0.0188494236 |
| CI = confidence interval; a/pCb = anterior/posterior cerebellum; GP = globus pallidus; H = Heschl’s gyrus; MG = medial geniculate thalamic nucleus; pAC = posterior auditory cortex; pIFS = posterior inferior frontal sulcus; preSMA = presupplementary motor area; pSTG = posterior superior temporal cortex; PT = planum temporale; SMA = supplementary motor area; VA = ventral anterior thalamic nucleus; VL = ventral lateral thalamic nucleus; vMC = ventral motor cortex; vPMC = ventral premotor cortex; VPM = ventral posterior medial thalamic nucleus; vSC = ventral somatosensory cortex. | | | | | |
|  |  |  |  |  |  |
