# Supplementary figures and images for: Differential involvement of feedback and feedforward control networks across disfluency types in adults who stutter: Evidence from resting state functional connectivity
Source: PLoS One. 2025 Sep 26;20(9):e0333205. doi: 10.1371/journal.pone.0333205 (PMC12468764; doi:10.1371/journal.pone.0333205)

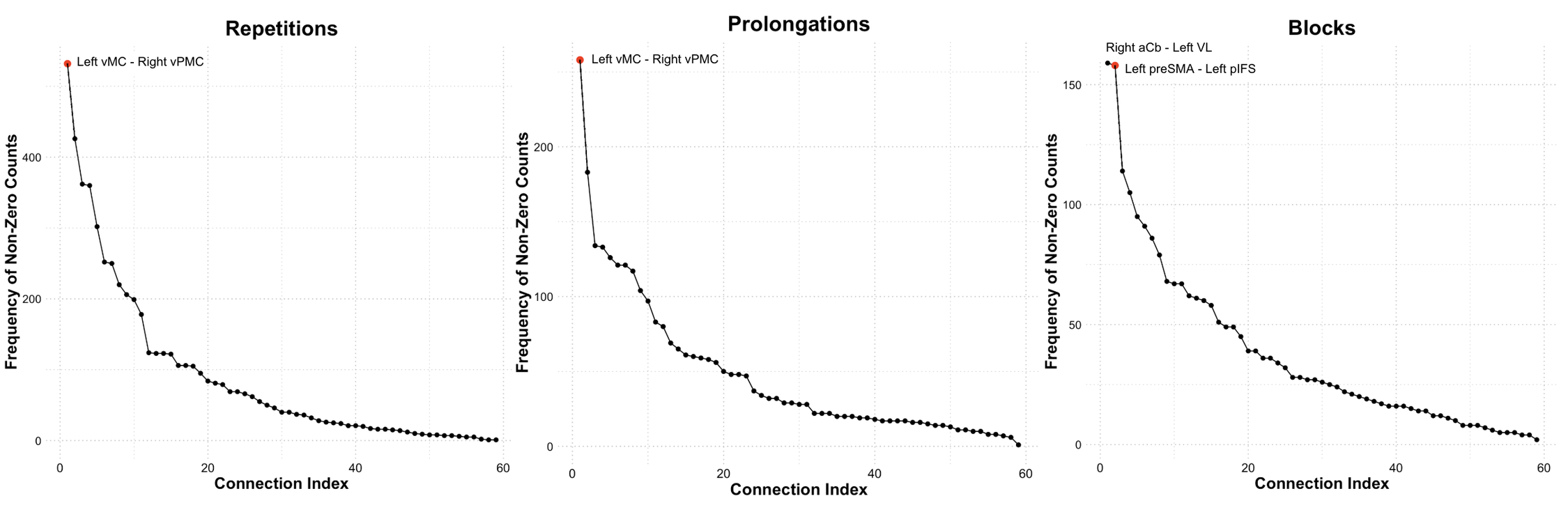

Supplement: Fig S1 — Red point = cutoff used to distinguish stable from unstable predictors, identified as the last variable before the largest drop in selection frequency; aCb = anterior cerebellum; pIFS = posterior inferior frontal sulcus; preSMA = presupplementary motor area; VL = ventral lateral thalamic nucleus; vMC = ventral motor cortex; vPMC = ventral premotor cortex. (TIF) [file pone.0333205.s003.tif]
