# Supplementary material for: Differential involvement of feedback and feedforward control networks across disfluency types in adults who stutter: Evidence from resting state functional connectivity
Source: PLoS One. 2025 Sep 26;20(9):e0333205. doi: 10.1371/journal.pone.0333205 (PMC12468764; doi:10.1371/journal.pone.0333205)
Supplement: Attachment — Submitted filename: ResponseLetter_PONE_R0_2025 (08.05.25).docx [file pone.0333205.s004.docx]

Dear Dr. Ning and our Reviewer,

My co-authors and I thank the editor and the reviewer for the insightful comments and suggestions regarding the original submission. We addressed all critiques to the best of our ability, hopefully in the direction that you and the reviewers envision. Thank you again for your thoughtful feedback. We appreciate the effort and time that went into reviewing this manuscript.

Below we address each critique from the reviewers.

***R1a: In line with the appropriate reference to the heterogeneity of the stuttering experience and its overt presentation, can the authors provide justification for their claims in determining the mechanisms affected among the subtypes of adults who stutter in this study, and whether this study was successful in contributing to a more unified understanding of stuttering?***

**RESPONSE:** Thank you for the insightful comment highlighting the complexity of stuttering and the importance of grounding mechanistic interpretations in the context of heterogeneity. We have updated the first paragraph of the discussion section to reflect this (and have also emphasized this point again in section 4.4):

While we hypothesized that repetitions and prolongations may reflect greater reliance on FB-based corrective mechanisms and that blocks may stem from disruptions in FF-based initiation mechanisms, our findings suggest only partially distinct connectivity patterns across disfluency types. Rather than implying entirely separate neural pathways, the results point to overlapping yet differentially weighted contributions of FB and FF systems, which may help explain the diversity of symptom expression in AWS.

***R1b: In the Introduction, please elaborate on the value of the proposed mechanistic understanding of stuttering to the success of treatment management and outcomes.***

**RESPONSE:** We have elaborated on the value of a mechanistic understanding of stuttering to treatment in the introduction (however, we have also expanded upon this more in the discussion as well):

Our goal is to explore potential associations that could guide future research into more targeted and individualized models of stuttering. For example, by identifying how specific neural control systems contribute to different disfluency types, we may eventually be able to tailor therapies that target the dominant mechanisms affecting each individual. However, we do not aim to make definitive conclusions at this stage, but rather to identify trends that might warrant further investigation in subsequent studies.

***R1c: The authors may consider revising what is deemed as the typical characterization of stuttering, and what are overt speech characteristics.***

**RESPONSE:** Thank you for this important suggestion. We have updated the introduction to address this point:

Finally, while in this study we focused on overt speech disruptions, we acknowledge that these behaviors do not capture the full complexity of the stuttering experience. However, focusing on these observable features provides a quantifiable and reproducible starting point for identifying potential neural mechanisms, which can later be expanded to encompass the broader phenomenology of stuttering.

***R1d: Please consider also revising the use of the term “abnormalities” in reference to feedback and feedforward processing, with distinctions or differential behavioural strategies associated with neurodevelopmental differences in stuttering.***

**RESPONSE:** We have changed the terminology to be “differences” rather than “abnormalities”.

***R1e: Consider also revising the use of the term “controls” in section 1.2.***

**RESPONSE:** We have replaced “controls” with “neurotypical speakers”.

***R1f: In the second sentence of section 1.4, please clarify that the resting state functional connectivity patterns of persons with persistent stuttering are being investigated.***

**RESPONSE:** We have clarified that we are referring to the resting state functional connectivity patterns of individuals with persistent stuttering. We have also rewritten the sentence to be more concise and straightforward.

***R1g: While describing the advantages of analysing resting state functional connectivity patterns, it would be helpful if the authors also outline the potential disadvantages, if any, when task-based functional activation in persons who stutter is interpreted.***

**RESPONSE:** Thank you for this suggestion. We have added potential disadvantages with task-based functional activation in persons who stutter:

Furthermore, resting-state functional connectivity analysis circumvents challenges commonly encountered in task-based studies of stuttering, such as difficulty in eliciting spontaneous disfluencies under controlled experimental conditions and increased motion artifacts during speech production tasks.

***R1h: When stating that the hypotheses regarding feedback and feedforward control are based on the aforementioned literature, please recite these specific studies for the reader.***

**RESPONSE:** We have recited the specific studies we were referencing in the first and second paragraphs of section 1.4.

***R1i: In addition, when linking different stuttering behaviors to the feedback and feedforward control networks, could the authors specify whether they expected network connectivity to be increased or decreased?***

**RESPONSE:** We had specified that we expect repetitions and prolongations will be primarily associated with increased connectivity in the FB control network, reflecting an overreliance on FB mechanisms, while blocks will be primarily associated with reduced connectivity in the FF control network, reflecting weakened FF mechanisms.

***R1j: The authors state that these findings could guide future research. Could they specify potential areas of further investigation?***

**RESPONSE:** We have briefly updated the introduction to elaborate on potential areas of further investigation (i.e., more targeted and individualized models of stuttering). We have expanded on these areas of investigation in the discussion section.

***R1k: Was clinical diagnosis conducted in the classification of these participants, or did this rely on self-disclosure alone? If the latter, the limitations and potential confound of this should be addressed.***

**RESPONSE:** Thank you for this important question. While participants were initially classified based on self-identification as people who stutter during a structured screening interview, each participant's speech was subsequently evaluated by a licensed SLP who is board certified in fluency. The SLP analyzed each speech sample to calculate total syllables, total stuttered syllables, and disfluency subtypes. This professional evaluation served to verify the presence and nature of stuttering in each participant, providing a level of clinical validation beyond self-report. We believe this procedure strengthens confidence in the classification of participants and mitigates the concern regarding reliance on self-disclosure alone.

***R1l: For Table 1, please explain each of the abbreviations used within the table, as well as revising the punctuation in the first sentence of the caption, and stating the total number of syllables elicited during speech testing.***

**RESPONSE:** Thank you for these suggestions. We have updated Table 1 with these edits.

***R1m: Please state the literature used to categorize the stuttering behaviors and guide the coding procedures.***

**RESPONSE:** We have updated the methods section to include the literature used to categorize the stuttering behaviors and guide the coding procedures.

***R1n: Please state the training and/or qualifications of the raters included in the reliability analysis.***

**RESPONSE:** We have included that the three raters in the reliability analysis consisted of the original rater (i.e., SLP who is a board-certified fluency specialist) and two additional SLPs.

***R1o: In section 2.4, please cite the validation studies for the CONN toolbox, as well as referenced support for the application of both surface-based and volumetric approaches for the analysis of functional connectivity between ROIs.***

**RESPONSE:** We have updated section 2.4 to include citations for the validation studies for the CONN toolbox and referenced support for the application of both surface-based and volumetric approaches for the ROI analysis.

***R1p: Could the authors please also cite support for their approach to bootstrapping?***

**RESPONSE:** We have updated the methods section to include the citation for our approach to bootstrapping.

***R1q: With repetitions and prolongations making up such a small proportion of the syllables used for analysis, along with the reported variability of all disfluency types among participants, could the authors speak to the limitations of this in interpreting their results?***

**RESPONSE:** Thank you for this important point. We have now addressed this explicitly in the limitations section:

Fifth, although repetitions and prolongations were analyzed alongside blocks, these disfluency types made up a relatively small proportion of the total syllables produced. Given the reported variability in disfluency patterns across participants, this imbalance may limit the interpretability of neural associations specific to those less frequent disfluency types. Future studies with larger sample sizes and more balanced disfluency distributions could better support comparisons across disfluency types.

***R1r: Figure 1: By visual inspection, it appears that the left medial geniculate thalamic nucleus and left Heschl’s gyrus show similar magnitude for blocks to that for prolongations. Can the authors report on the significance of this association and clarify why this is not discussed? This may speak to the authors’ recognition that individual differences or heterogeneity of resting state functional connectivity may be limited in providing unique or distinct neural characteristics of developmental stuttering.***

**RESPONSE:** Thank you for this thoughtful observation. Upon revisiting our feature selection process using an updated elbow method, we found that the connection between the left medial geniculate thalamic nucleus and left Heschl’s gyrus for prolongations did not meet the threshold for inclusion in the final feature set. However, we agree that its coefficient magnitude remains noticeably larger than several others that were retained, and thus it may carry meaningful signal despite not reaching our data-driven cutoff. We concur with the reviewer’s broader point that this pattern underscores a potential limitation of resting-state functional connectivity in fully capturing unique or distinct neural signatures of developmental stuttering. It highlights the importance of considering individual variability and suggests that complementary neural or behavioral markers may be necessary to more precisely characterize subtypes or symptom dimensions within this population.

***R1s: The literature that is cited to support the contribution of the anterior cerebellum in optimizing the accuracy of speech motor commands is primarily focused on manual motor coordination. Can the authors clarify this within the text and speak to the generalizability of these previous research findings to speech motor coordination?***

**RESPONSE:** Thank you for bringing up this important point. We have clarified in the text that the literature we cited is primarily focused on manual coordination and have elaborated on the generalizability of this research to findings in speech motor coordination:

While much of the supporting literature has focused on the cerebellum’s role in manual motor coordination (Diedrichsen et al., 2005; Grafton et al., 2008; Penhune & Doyon, 2005), there is growing evidence that these same predictive control mechanisms generalize to speech motor control. Similar to limb control, speech movements rely on the cerebellum to generate internal models that anticipate the sensory consequences of motor commands, allowing for the smooth and timely execution of complex motor sequences (Runnqvist et al., 2021). In speech, this function is particularly critical given the rapid and precise coordination required across multiple articulators. Furthermore, neuroimaging studies have shown overlapping cerebellar regions involved in both limb and speech tasks, suggesting shared computational principles (Manto et al., 2012; O’Reilly et al., 2010). Therefore, disruptions in cerebellar-thalamic connectivity, such as reduced connectivity between the right aCb and left VL, may reflect impairments in these predictive mechanisms, leading to difficulties initiating or coordinating speech movements. This may help explain our finding that reduced connectivity in this FF circuit is associated with an increased frequency of blocks.

***R1t: Section 4.4 highlights the fact that not one type of stuttering behavior is specific to an individual’s experience and presentation of stuttering, with the overlap of associations between ROI connectivity also warranting further emphasis.***

**RESPONSE:** We completely agree that these are important points, and we have tried our best to further emphasize them in section 4.4:

Stuttering is a multifaceted disorder with variable manifestations across individuals. While this study explored the hypothesis that repetitions, prolongations, and blocks may be differentially associated with FB and FF control systems, our findings do not suggest clean distinctions between the connectivity patterns supporting these systems. Rather, the results point to considerable overlap in the neural pathways associated with different disfluency types, indicating that these control mechanisms likely interact in complex and non-exclusive ways.

Notably, it is rare for individuals to exhibit only one type of disfluency; most present with a combination of repetitions, prolongations, and blocks. This behavioral overlap is reflected in the shared involvement of several ROI connections across disfluency types, suggesting that stuttering behaviors may arise from a common set of neural mechanisms modulated by individual differences in neural dynamics or control system balance. Instead of representing entirely distinct processes, repetitions, prolongations, and blocks may reflect different manifestations of disruptions within a shared speech motor network.

Thus, this study does not argue for entirely separate neural origins of disfluency types, but rather offers an initial step toward identifying network-level variations that may help explain individual differences in stuttering expression. Future work with larger samples and symptom-specific subgrouping may help clarify the extent to which certain neural pathways confer vulnerability to particular disfluency profiles.

***R1u: In discussing the limitations of using resting state functional connectivity, the authors may consider further highlighting the need to measure individuals’ experiences of anxiety, stress, attention, and linguistic proficiency.***

**RESPONSE:** Thank you for bringing this to our attention. We have updated the limitations section to highlight the need to measure these important variables:

Moreover, internal states such as anxiety, stress, attention, and linguistic proficiency likely contribute to stuttering behaviors and may modulate neural network activity in ways not captured by resting-state measures. Future studies would benefit from incorporating direct assessments of these individual factors to better contextualize the neural mechanisms associated with disfluency patterns and improve the interpretability of resting-state connectivity findings.

***R1v: It is important to note that the findings do not cleanly distinguish or differentiate the connectivity patterns that support feedback and feedforward control. The interpretation of results may be more nuanced than the authors have described.***

**RESPONSE:** Thank you for this important point. We have updated section 4.4 in the discussion to better reflect this nuance (see our response to R1t).

***R1w: In discussing the potential benefits of targeted neuromodulation or pharmacology, could the authors briefly summarize the findings from studies of neuromodulatory and pharmacological effects in this population?***

**RESPONSE:** Thank you for this suggestion. We have updated the discussion section to include a brief summary of findings from studies of neuromodulatory and pharmacological effects in adults who stutter:

Prior studies have reported increased fluency following stimulation to speech motor areas, including inferior frontal gyrus and supplementary motor area (Chesters et al., 2018; Garnett et al., 2019). Similarly, pharmacological interventions have targeted dopaminergic pathways, given the proposed role of dopamine dysregulation in stuttering (Maguire et al., 2000; Wu et al., 1997).

**AEa: *Please add page numbers and line numbers.***

**RESPONSE:** We have added page numbers and line numbers.

**AEb: *Table 1: Please elaborate on how the SSI-4 was measured for each participant. In addition to the percentages, please also provide the raw counts and the total number of syllables for each cell.***

**RESPONSE:** We have updated the methods section to include additional details about how the SSI-4 was measured for each participant. We have also updated Table 1 to include the raw count and the total number of syllables for each cell.

***AEc: Sect 2.2, Para 1, Line 4: “The topic of the conversational samples” -> Make it clear that this is “in-person conversational samples”.***

**RESPONSE:** We have made this detail clear.

***AEd: Sect 2.2, Para 1: Your in-person conversational samples were, on average, twice as long as the phone conversational samples and the reading samples. I wonder why the task effect was not tested in the statistical model. Could it be that people who stutter tend to produce more stuttering behaviors as speech production length increases?***

**RESPONSE:** Thank you for this insightful comment. You are correct that the in-person conversational samples were longer on average than the phone and reading samples. Our primary focus in the current analysis was on comparing disfluency patterns across communication contexts, rather than investigating task effects related to sample length. We acknowledge that longer speech samples may provide greater opportunity for disfluencies to occur, and future work could more directly examine the relationship between sample duration and stuttering frequency by including sample length as a covariate or testing for interaction effects. We have added this point as a potential limitation and future direction in the revised manuscript:

Fourth, while we examined disfluency patterns across different communication contexts, we did not model the potential influence of sample length or task duration on disfluency frequency. For example, in-person conversational samples tended to be longer than phone or reading samples, which may have increased opportunities for disfluencies to occur. Future analyses could include sample length as a covariate to better isolate effects related to context versus duration.

***AEf: Sect 2.2, Para 2, Line 8: “…involves the occurrence of two or more different stuttering types in a single instance” -> What exactly do you mean by “single instance”? Is it a sentence, an utterance, a phrase, a word, or something else?***

**RESPONSE:** We have clarified in the manuscript that we were referring to a “single stuttering moment”.

***AEg: Sect 2.2, Para 2, Lines 9-10: “…the stutter was classified according to the dominant disfluency type within that occurrence” -> What if there was a tie between disfluency types? How was the category assigned in that case?***

**RESPONSE:** Thank you for this thoughtful question. We have clarified our decision-making for ties in disfluency types in the methods section:

In instances where there was a tie between disfluency types within a single stuttering event, the classification was determined based on the first disfluency to occur in the sequence. This decision was made to ensure consistency across coding and to avoid arbitrary assignment when multiple types co-occurred.

***AEh: Sect 2.5, Para 2, Lines 9-10: “The results did not indicate a significant effect, suggesting that further normalization across sites was not necessary.” -> Please provide the Levene’s test statistics, as these are required to support this claim.***

**RESPONSE:** We agree that the Levene’s test statistics are required to support this claim, and we have included a table with the results of Levene’s tests assessing heteroscedasticity due to inter-site variance (scanner effects) (Table S1 in Supplementary Material).

***AEi: Sect 3.2, Para 1, Lines 7-9: “All predictors showed statistically significant effects, as their CIs did not include zero, indicating consistent and non-random relationships across the resampled datasets.” -> Please provide a summary table or a forest plot to support this claim.***

**RESPONSE:** We have included a summary table with coefficient estimates, standard errors, and 95% confidence intervals of connections with statistically significant effects identified by each LASSO regression (Table S2 in Supplementary Material).

***AEj: Figure 1 caption: You may need to explain what the black rectangles (the selected variables after applying the elbow method?) represent, both in the figure caption and in the main text.***

**RESPONSE:** Thank you for this suggestion. We have clarified both in the figure caption and in the main text what the black rectangles represent (i.e., the selected predictors identified using the elbow method, reflecting those with the most consistent influence).

***AEk: Sect 3.2, Para 2, Lines 1-4: “The stability selection method was then conducted to identify the most consistently influential predictors across the 1000 iterations. Lastly, the elbow method was applied to determine the cutoff point for the predictors that had non-zero coefficients in the highest number of model iterations.” -> Please provide the elbow plot for each type (repetition, prolongation, and block).***

**RESPONSE:** Thank you for this suggestion. We have included elbow plots for each disfluency type (repetition, prolongation, and block) (Figure S1 in Supplementary Material). We also wish to clarify that the algorithm used to identify the most consistently selected features is based on detecting the first prominent drop in selection frequency, rather than the traditional “elbow point.” This approach reflects a more conservative threshold, prioritizing the stability and reproducibility of selected predictors. We have updated the methods section accordingly to explain this distinction:

To determine a cutoff for variable inclusion based on stability selection frequencies, we used a data-driven heuristic inspired by scree plot and elbow methods. Specifically, we identified the index at which the first prominent drop in selection frequency occurred among predictors, ranked by their selection count across bootstrap samples. This point was interpreted as the last relatively stable variable before the onset of a sharp decline, indicating a transition from highly stable to less consistently selected predictors. Although this approach does not provide formal control of false discoveries, it offers a pragmatic balance between sparsity and reproducibility in the context of exploratory feature selection. Compared to traditional elbow methods, our "first drop" heuristic imposes a more conservative criterion for feature selection. Rather than identifying the point of diminishing returns, it targets the onset of instability in selection frequencies, yielding a smaller but more stable set of predictors. This stricter threshold prioritizes reproducibility over model complexity, aligning with our goal of identifying robust, high-confidence variables.

***AEl: Sect 4.2, Para 4 and Figure 4: There is a phonological content buffer in the planning loop of the GODIVA model. I’m curious whether the participants had more difficulty initiating onset consonants or the following vowels (e.g., in a CV syllable). Given that vowels are generally more prominent or dominant in a syllable, did they experience more blocks during vowel production compared to consonant production?***

**RESPONSE:** Thank you for this insightful question. While the GODIVA model’s phonological content buffer does suggest possible distinctions in the planning and initiation of consonants versus vowels, we did not perform a phoneme-level analysis distinguishing whether blocks occurred more frequently on consonants or vowels. As such, this question falls beyond the scope of the current study. However, examining the phonetic locus of disfluencies, particularly in relation to syllable structure, would be a valuable future direction for exploring how deficits in speech planning and initiation may manifest in different disfluency types.

***AEm: Please check the apostrophes and quotes throughout the entire manuscript, as some appear to have inconsistent formatting (possibly from copying and pasting from other resources). For example, see Line 8 of Section 1.4, Lines 8 and 10 of Section 2.3, Line 10 of Section 2.4.2, and Line 10 of Section 5.***

**RESPONSE:** Thank you for this observation. We have corrected all apostrophes and quotes.
