# Supplementary material for: Differential involvement of feedback and feedforward control networks across disfluency types in adults who stutter: Evidence from resting state functional connectivity
Source: PLoS One. 2025 Sep 26;20(9):e0333205. doi: 10.1371/journal.pone.0333205 (PMC12468764; doi:10.1371/journal.pone.0333205)
Supplement: Attachment — Submitted filename: ResponseLetter_PONE_R1_2025 (09.04.25).docx [file pone.0333205.s005.docx]

Dear Dr. Ning and our Reviewer,

My co-authors and I thank the editor and the reviewer for the insightful comments and suggestions regarding the revised submission. We addressed all critiques to the best of our ability, hopefully in the direction that you and the reviewer envision. Thank you again for your thoughtful feedback. We appreciate the effort and time that went into reviewing this manuscript.

Below we address each critique.

***R1a: Abstract: Page 2, line 17: The authors note in their discussion of results that their “findings do not suggest clean distinctions between the connectivity patterns supporting these systems”. Yet, in the Abstract, they report the identified neural underpinnings as being “distinct”. For consistency, please revise this interpretation of distinctness throughout the manuscript, including the later section on Theoretical and clinical implications.***

**RESPONSE:** We appreciate this point. We have updated the Abstract to reflect this change and have also revised the interpretation of distinctness throughout the manuscript.

***R1b: Introduction: Page 3, lines 2-3: In the interest of recognizing the multifaceted experience of stuttering, the authors may consider describing the “overt speech behaviors” of stuttering as being generally characterized by repetitions, prolongations and blocks.***

**RESPONSE:** Thank you for this suggestion. We have updated that sentence to read: Stuttering is generally characterized by overt speech behaviors such as repetitions (i.e., repeated sounds or syllables), prolongations (i.e., extended duration of a sound), and blocks (i.e., temporary stoppage of airflow or vocalization).

***R1c: Introduction: Page 7, line 15: The authors may wish to vary the wording for “capturing” in this sentence, which appears twice.***

**RESPONSE:** Thank you for catching this. We have replaced the second instance of “capturing” with “reflecting”.

***R1d: Methods: Page 9, line 4: If interpreted correctly, the term “social context” may need to be pluralized here.***

**RESPONSE:** Thank you. We have made this change.

***R1e: Methods: Page 11, line 12: It is still unclear how moments of disfluency were classified according to the dominant disfluency type. Was this dominance judged by the first disfluency type to occur or measured by the length of the disfluency type? The issue of mixed disfluencies may need to be addressed here, as well as how the potential dominance of perceptible blocks may have also influenced their prevalence within participants’ speech samples. This is again noted below.***

**RESPONSE:** Thank you for this clarification. We have updated the Methods section to be more clear about how we judged dominance and address how potential dominance of perceptible blocks may have influenced their prevalence.

“Dominance” was operationalized as the feature occupying the greater proportion of the stuttering moment. In rare cases where no type was clearly dominant, the classification was determined based on the first disfluency to occur in the sequence. Because blocks are often perceptually salient, coders were trained to apply this rule conservatively to avoid over-classification of blocks when they occurred in combination with repetitions or prolongations. This approach ensured that mixed disfluencies were consistently classified while minimizing the risk that blocks would be disproportionately coded as dominant.

***R1f: Methods: Page 12, line 23: With two citations referenced in support of CONN toolbox validation, the authors may wish to note that this has been “validated in the field” and not “extensively”, unless specific validation studies can be cited.***

**RESPONSE:** We agree and have removed the term “extensively”.

***R1g: Discussion: Page 26, lines 4-9: Here it is stated that “researchers have proposed that stuttering may evolve over the lifespan, progressing from relatively effortless repetitions to more tense, rapid, or irregular repetitions, prolongations, and eventually blocks” which may reflect “an increasing awareness of, and reactions to, stuttering, as well as attempts to exert control over speech”. Can the authors identify additional support of these claims in the literature? The following report on age effects on disfluency types may be considered here: Staróbole Juste, F., & Furquim de Andrade, C. R. (2011). Speech disfluency types of fluent and stuttering individuals: age effects. Folia Phoniatrica et Logopaedica, 63(2), 57-64.***

**RESPONSE:** Thank you for this suggestion. We have added this fascinating report as evidence to support these claims in the Discussion section.

Evidence from developmental studies of stuttering further supports this view, showing age-related shifts in disfluency patterns, with older individuals tending to exhibit more blocks relative to repetitions.

***R1h: Discussion: Page 26, lines 10-14: Please state which models emphasize the emotional and cognitive responses to stuttering. References to alternative and/or complimentary models, frameworks and/or theories should be included here.***

**RESPONSE:** This is an important point. We have updated the Discussion section to include the references for these alternative models.

Indeed, while DIVA and GODIVA primarily focus on disruptions in FB and FF control circuits, other models emphasize emotional and cognitive responses to stuttering. For example, the Covert Repair Hypothesis (114) attributes disfluencies to internal monitoring and error correction in speech planning, while the Dual Diathesis-Stress Model (115) highlights how emotional reactivity, self-regulation, and cognitive load shape stuttering expression. These alternative perspectives suggest additional involvement of networks related to self-monitoring, emotional regulation, and executive control, complementing sensorimotor accounts and underscoring the multifactorial nature of stuttering.

***R1i: Discussion: Pages 26-27, lines 22-10: As mentioned earlier, the authors’ interpretation that “repetitions, prolongations, and blocks may reflect different manifestations of disruptions within a shared speech motor network”, as opposed to distinct neural mechanisms, could be more consistently reported throughout the manuscript.***

**RESPONSE:** We agree and have updated the manuscript to be more consistent with this interpretation.

***R1j: Discussion: Page 27, lines 13-15: In considering potential follow-up investigations and future research on this topic, potential age or developmental effects on disfluency types, their neural associations, and their predominance could be listed here.***

**RESPONSE:** Thank you for this suggestion. We have updated the Limitations section to include these variables.

***R1k: Discussion: In addition, the limitations of disfluency type classification should be considered, particularly when mixed or clustered disfluencies arise. In the current investigation, consideration could also be given to how the disfluency type of “blocks” may be more perceptible or perceived as being more dominant, consequently reducing the perceived prevalence of repetitions or prolongations – particularly if these behaviors were more subtle. Arguments against disfluency-type classification have also been made: Einarsdóttir, J., & Ingham, R. J. (2005). Have disfluency-type measures contributed to the understanding and treatment of developmental stuttering?. American Journal of Speech-Language Pathology, 14(4), 260-273. + Bothe, A. K. (2008). Identification of children’s stuttered and nonstuttered speech by highly experienced judges: Binary judgments and comparisons with disfluency-types definitions. Journal of Speech, Language, and Hearing Research, 51(4), 867-878.***

**RESPONSE:** We appreciate this point and have updated the Limitations section to reflect this important suggestion.

Sixth, classification of disfluency type introduces its own limitations, particularly when mixed or clustered disfluencies occur within the same moment. Although we coded by dominant disfluency type, blocks may have been more perceptible and thus more likely to be classified as dominant, potentially underestimating the prevalence of repetitions or prolongations when they co-occurred. This perceptual weighting could influence both frequency estimates and their associated neural patterns. Moreover, debates remain about the overall utility of disfluency-type classification. Some scholars have questioned whether such measures meaningfully advance our understanding or treatment of stuttering (114,115). These critiques underscore the need for caution in interpreting findings tied to disfluency categories and illustrate the importance of developing complementary analytic approaches (e.g., multidimensional or continuous measures of stuttering severity).

***R1l: Supplementary Materials: Table S2: It appears that blocks had the most statistically significant effects, followed by repetitions, and prolongations having fewer statistically significant effects on resting state connections. The relevance of these findings could be discussed within the reporting of results, as well as their potential implications in the discussion section.***

**RESPONSE:** Thank you for bringing this to our attention. We have updated the Results section to discuss these results:

Notably, although blocks were associated with fewer overall predictors than repetitions or prolongations, the predictors that did emerge for blocks showed larger effect estimates and required greater regularization, suggesting stronger and potentially more robust brain–behavior relationships. By contrast, prolongations exhibited fewer and weaker associations, consistent with their relatively lower prevalence in the behavioral data.

We have also updated the Discussion section regarding their potential implications:

Of note, blocks also showed the strongest and most robust associations overall compared to repetitions and prolongations, consistent with their greater behavioral prevalence in our sample. This pattern suggests that blocks may be more tightly coupled to identifiable neural circuitry, whereas repetitions and especially prolongations may reflect more subtle or variable connectivity differences.
